# Layer‐by‐layer soft‐tissue effects on flexion–extension‐dominant passive ex vivo limb joint ROM in quadrupedal mammals: An anatomical contribution to a morphofunctional framework

**DOI:** 10.1111/joa.70207

**Published:** 2026-06-30

**Authors:** Paul Medina‐González, Valentina Bernal‐Fernández, Rodrigo Arancibia‐Müller, Pedro Aburto‐Valdebenito, Marcelo Gómez‐Jaramillo

**Affiliations:** ^1^ Departamento de Kinesiología, Facultad de Ciencias de la Salud Universidad Católica del Maule Talca Chile; ^2^ Laboratorio de Morfología Comparada, Instituto de Farmacología y Morfofisiología, Facultad de Ciencias Veterinarias Universidad Austral de Chile, Campus Isla Teja Valdivia Chile

**Keywords:** comparative functional morphology, ex vivo biomechanics, joint range of motion, limb posture, morphofunctional framework, soft‐tissue effects

## Abstract

Functional reconstructions of extinct mammals often infer joint mobility from osteological geometry, yet the mobility envelope in life emerged from bones embedded within a layered soft‐tissue system. More broadly, passive joint mobility provides an anatomical bridge between osteological form, soft‐tissue constraints, and biological movement. Ex vivo range‐of‐motion (ROM) datasets that explicitly partition soft‐tissue contributions across multiple joints and postural types remain scarce for mammals. Here, we quantify layer‐by‐layer effects on flexion–extension‐dominant passive ex vivo limb joint ROM across six major limb joints (shoulder, elbow, wrist [carpal joint], hip, knee [stifle] and ankle [hock]) in four quadrupedal mammals spanning differing postures: rabbit (*Oryctolagus cuniculus*), chilla fox (*Lycalopex griseus*), pig (*Sus scrofa domestica*) and pudu deer (*Pudu puda*) (one specimen per taxon). ROM was measured sequentially under four anatomical conditions that progressively isolate tissue contributions: intact (S+M+CL+O), myofascial (M+CL+O), capsulo‐ligamentous (CL+O) and osteology‐only (O). Three trained evaluators passively moved each joint to maximal flexion and extension endpoints. Endpoints were recorded using calibrated photographs, and joint angles were quantified via vector‐based analysis by a single experienced assessor. Between‐evaluator dispersion was summarised using SD and coefficient of variation (CV%). Passive ROM did not increase monotonically with tissue removal. Instead, trajectories were strongly joint‐ and taxon‐specific, with frequent intermediate‐condition maxima and, in several distal joints, marked reductions in the osteology‐only condition after capsulo‐ligamentous removal. The wrist showed the most pronounced non‐monotonicity, commonly peaking at CL+O and decreasing sharply in O, in some cases, to values below the intact state. By contrast, proximal joints more often exhibited large net expansions across the dissection sequence, although the condition producing maximal ROM varied among taxa and joints. Measurement dispersion also varied by joint and condition, tending to be higher in intact states and in joints that were more complex to manipulate consistently, especially distal joints, consistent with less sharply defined passive endpoints when multiple layers contribute distributed resistance. These results indicate that osteology‐only ROM is not a reliable upper bound on biologically feasible flexion–extension‐dominant passive motion, because periarticular tissues can both constrain excursion and stabilise alignment within multi‐element joint complexes. Accordingly, layer‐resolved ROM series provide an empirical anatomical line of evidence that can guide sensitivity analyses in musculoskeletal reconstructions and support cautious calibration of morphofunctional spaces for movement interpretation, including future palaeobiological applications.

## Graphical abstract

Layer‐by‐layer passive ex vivo assessment of flexion–extension‐dominant limb joint ROM across rabbit, pudu deer, chilla fox and pig revealed joint‐, tissue‐ and taxon‐specific responses to progressive tissue removal. Proximal joints often showed increasing ROM, whereas distal joints often showed non‐monotonic responses. These data provide tissue‐informed bounds linking osteological form, passive ex vivo mobility and active in vivo movement within a morphofunctional framework.

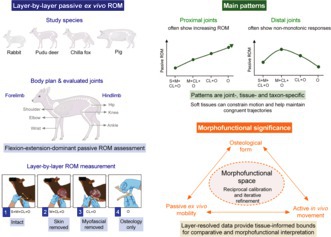

## 1 INTRODUCTION

Functional reconstructions of extinct mammals and other fossil vertebrates often begin by inferring joint mobility from osteological geometry and by assuming that articular congruence and bony stop angles define the limits of limb range of motion (ROM) (Senter & Robins, 2005; Pierce et al., 2012; Jannel et al., 2019; Richards et al., 2021; Manafzadeh et al., 2024; Benton & Rayfield, 2026). This osteological starting point has yielded valuable insights, yet it can underplay the mechanical role of soft tissues, including ligaments, joint capsules, tendons, fascia, and skin, whose material properties, three‐dimensional arrangement and thickness can either constrain or facilitate movement in taxon‐ and joint‐specific ways (Arnold et al., 2014; Broyde et al., 2021; Burgio et al., 2023; Demuth et al., 2025; Manafzadeh & Padian, 2018). Joint mobility is therefore best understood as an intermediate anatomical level between osteological form and biological function, rather than as a direct read‐out of bone geometry alone (Manafzadeh, 2023). Experimental and modelling work shows that joint torques and effective muscle moment arms are modulated by the presence and condition of soft tissues. For example, variation in cartilage thickness and articular shape can shift centres of rotation and alter load transmission by relocating contact points (Brassey et al., 2017; Wiseman et al., 2022). Consequently, the same osteological template can express divergent functional outcomes depending on its soft‐tissue envelope.

This issue is relevant to palaeobiology because fossils preserve articular geometry whereas the realised joint envelope in life is bounded by an integrated system of extrinsic and intrinsic soft tissues. The mechanical contributions of these tissues can be substantial and they vary across taxa and joints. Comparative work highlights that ligament and capsule properties span orders of magnitude in stiffness and elastic moduli, reflecting divergent loading regimes, composition and specialisation (Burgio et al., 2023). Moreover, measured properties depend on protocol choices such as strain rate and preconditioning, which can bias parameterisation when generic values are imported into functional interpretations (Broyde et al., 2021; Burgio et al., 2023). In keeping with the broader point that biological performance emerges from non‐linear interactions among components, joint function is shaped by the integrated soft‐tissue envelope, not only by the bones (Lauder, 1995). This point is especially important because modern palaeobiological reconstructions increasingly distinguish osteological mobility, passive soft‐tissue‐bounded mobility and in vivo kinematics as related but non‐equivalent levels of inference (Manafzadeh et al., 2021, 2024).

Despite these concerns, ex vivo ROM assessments in mammals remain methodologically heterogeneous and taxonomically sparse, particularly for designs that explicitly partition the contribution of successive tissue layers. In other clades, repeated‐measures dissections performed layer by layer show that mobility is highly sensitive to periarticular tissues and that controlled manipulation can be reproducible between operators, even when absolute angles vary among trials (Hutson & Hutson, 2012). In the dinosaur extant phylogenetic bracket (EPB), Hutson & Hutson first tested elbow mobility in *Struthio camelus* and *Alligator mississippiensis*, and subsequently extended the same repeated‐measures logic to shoulder mobility in *Alligator mississippiensis* and wrist mobility in the avian–crocodylian bracket (Hutson & Hutson, 2012, 2013, 2014). Together, these studies show that soft‐tissue effects are not uniform across the limb, but vary according to joint architecture, tissue condition, and the anatomical complexity of the joint being manipulated.

Prior work further indicates that these tissue effects can markedly shift ex vivo mobility envelopes and are therefore relevant when defining biologically plausible bounds on joint motion in fossil reconstructions (Arnold et al., 2014; Manafzadeh & Padian, 2018). In mammals, however, comparable layer‐resolved datasets spanning multiple limb joints and postural types remain uncommon, even though periarticular structures, including deep fascia, can impose measurable mechanical constraints on joint behaviour (Chong & Davies, 2018). This gap limits the anatomical basis for comparing mobility estimates among taxa that differ in posture and locomotor strategy.

Over the last decade, joint mobility research has advanced substantially through biplanar imaging, XROMM, six‐degree‐of‐freedom pose‐space analyses, multi‐joint pose viability and musculoskeletal modelling (Bishop et al., 2021; Brocklehurst et al., 2025; Demuth et al., 2025; Herbst et al., 2022; Manafzadeh & Gatesy, 2021; Pierce et al., 2012). These approaches have refined the reconstruction of coupled rotations and translations, long‐axis rotation, internal joint distance, and articular congruence. At the same time, their anatomical interpretation depends on assumptions about soft‐tissue geometry, material behaviour, and joint‐surface relationships. Layer‐resolved ex vivo experiments can therefore provide empirical tissue‐level evidence that helps ground, constrain and refine these higher resolution approaches. In mammals, such evidence remains limited, especially across multiple joints and differing postural types. This raises a central anatomical question: how do real mammalian soft‐tissue layers modify a repeatable flexion–extension‐dominant passive ROM trajectory under ex vivo conditions?

There is therefore a clear need for comparative, layer‐resolved experiments in mammals that quantify the contributions of integument and subcutaneous tissues, myofascia, and capsulo‐ligamentous structures to realised passive ROM under controlled handling and with explicit repeatability. Such experiments are anatomically valuable because dissection‐based manipulation can isolate tissue layers in a manner that is difficult to achieve in vivo, while generating interpretable effect sizes that can be used as empirically grounded constraints. More generally, these data can help to clarify when osteology‐only mobility estimates are likely to diverge from soft‐tissue‐bounded mobility (Broyde et al., 2021). They can also provide empirical soft‐tissue evidence for refining assumptions used in computational or in silico reconstructions, which necessarily require parameterisation of soft‐tissue behaviour, cartilage‐mediated spacing, and articular congruence.

Here, we present a descriptive, layer‐resolved assessment of passive flexion–extension‐dominant ROM and how it changes across successive tissue conditions. Rather than assuming monotonic expansion with tissue removal, we document joint‐ and taxon‐specific trajectories, including potential roles for capsulo‐ligamentous structures in both restricting excursion and maintaining alignment within multi‐element complexes. We also describe how patterns differ across joints and postural types, providing an anatomical basis for interpreting when osteology‐only mobility estimates may diverge from soft‐tissue‐bounded passive envelopes. To document these patterns, we quantify layer‐by‐layer effects on flexion–extension‐dominant ex vivo limb joint ROM across four extant quadrupedal mammals spanning differing postural categories and limb morphologies: rabbit (*Oryctolagus cuniculus*, medium‐bodied plantigrade), chilla fox (*Lycalopex griseus*, medium‐bodied digitigrade), pig (*Sus scrofa domestica*, large‐bodied unguligrade), and pudu deer (*Pudu puda*, medium‐bodied unguligrade). These taxa provide a comparative framework grounded in established postural classifications and their evolutionary transitions (Carrano, 1997; Kubo et al., 2019). Because postural and locomotor categories can behave as continua rather than rigid states, these descriptors are used here as comparative anatomical categories rather than deterministic predictors of movement (Carrano, 1999; Gambaryan, 1974).

The aims of this study are to identify comparative patterns of soft‐tissue effects by anatomical layer and joint, to provide empirically derived layer‐specific differences that link osteology‐only and soft‐tissue‐bounded passive ROM, and to deliver a repeatable ex vivo protocol for multi‐evaluator measurement in mammals. More broadly, this study contributes an anatomical ex vivo line of evidence to a morphofunctional framework in which morphology, passive mobility, and active movement can be integrated in future comparative and palaeobiological applications. Accordingly, the central research question is: how do successive soft‐tissue layers modify passive ex vivo flexion–extension‐dominant ROM across mammalian postural types, and how consistently do these effects vary among joints and taxa?

## 2 METHODS

### 2.1 Institutional permissions and specimen provenance

All work used cadaveric specimens donated to the Laboratorio de Morfología Comparada (Comparative Morphology Laboratory), Universidad Austral de Chile, for research and teaching. Access, handling and dissection were authorised by the Head of the Laboratory, and procedures followed the institution's policy for research on donated animal cadavers. For each specimen, provenance, storage history, dissected side and, when available, cause of death were recorded. Reporting was informed by relevant ARRIVE 2.0 items, adapted for ex vivo cadaveric experimentation, recognising that guidance specific to in vivo experimentation does not apply to cadaveric studies. Specimen metadata are provided in Table 1 and Supplementary File 1.

**TABLE 1:** Specimen metadata, handling and storage for the four mammals used in the layer‐resolved passive ROM assessments.

Species	Sex	Age class	Side dissected	Post‐mortem interval (hours)	Storage mode	Thawing time (hours)	Posture category	Body mass (kg; class)
*Oryctolagus cuniculus*	Male	Adult	Right	24	Refrigerated (4°C)	48	Plantigrade	2.5; Medium
*Pudu puda*	Female	Adult	Left	72	Frozen (single freeze–thaw cycle)	48	Unguligrade	10.0; Medium
*Lycalopex griseus*	Male	Adult	Left	72	Frozen (single freeze–thaw cycle)	72	Digitigrade	5.0; Medium
*Sus scrofa domestica*	Male	Juvenile	Left	12	Refrigerated (4°C)	72	Unguligrade	45.0; Large

*Note*: Specimen identity includes species, sex and age class. Sampling and handling variables include dissected side, post‐mortem interval, storage mode and thawing time. Biological factors include posture category and body mass, reported as broad contextual variables following previous morphofunctional operationalisations (Medina‐González, 2025, 2026). Body mass classes were based on literature‐derived species accounts (Pineda‐Muñoz et al., 2016), and limb posture categories follow Carrano (1997, 1999). Rabbit foot posture can vary with task and speed; it is classified here as plantigrade for comparative grouping. For each taxon, one forelimb and one hindlimb from the same individual were analysed.

### 2.2 Taxa, sampling design, and limb selection

We examined four extant quadrupedal mammals spanning differing limb postures (plantigrade, digitigrade and unguligrade): rabbit (*Oryctolagus cuniculus*), chilla fox (*Lycalopex griseus*), pig (*Sus scrofa domestica*), and pudu deer (*Pudu puda*). A juvenile alpaca (*Vicugna pacos*) hindlimb was used only for a repeatability pilot (see reliability indicators in Supplementary File 2).

For each taxon in the main comparison, one specimen was analysed (*n* = 1 per taxon) and a single limb was selected after visual inspection by an experienced laboratory dissector, prioritising preservation of periarticular tissues and absence of gross damage. Side selection is reported in Table 1.

### 2.3 Post‐mortem interval, storage, and handling

All specimens were processed within 1 week of death and measurements were performed after the expected window of rigour mortis to minimise bias from transient post‐mortem muscle stiffness. To limit degradation, limbs were stored either refrigerated at 4°C or frozen for approximately 1 week and subsequently thawed under refrigeration for 48 h prior to testing to ensure complete thawing while minimising decomposition, resulting in a single freeze–thaw cycle where applicable. During preparation and testing, tissues were kept moist by regular application of 0.9% saline. Laboratory temperature ranged from 20°C to 23°C, and ambient humidity was not controlled. No chemical fixation was applied. These handling criteria were selected to preserve native soft‐tissue mechanical behaviour as closely as possible during ex vivo testing. Post‐mortem interval (h), storage mode and thawing time are reported in Table 1.

### 2.4 Experimental overview and tissue‐layer definitions

Passive ROM was quantified sequentially across four anatomical tissue conditions using a flexion–extension‐dominant passive mobilisation protocol that progressively isolates the contribution of soft tissues, culminating in an osteology‐only condition.

In this study, passive ROM was defined as the range of joint motion elicited by external manual manipulation in the absence of active neuromuscular contraction, following standard biomechanical and clinical usage of passive joint motion (Kisner et al., 2018; Neumann, 2017; Norkin & White, 2016). In the present ex vivo context, this definition also aligns with the comparative concept of joint mobility as the set of configurations that a joint can passively assume (Manafzadeh, 2023).

Tissue conditions are abbreviated as follows:

1. Intact (S+M+CL+O): skin and subcutaneous tissues retained.
2. Myofascial (M+CL+O): skin and subcutaneous tissues removed; muscles, tendons, and fascia retained.
3. Capsulo‐ligamentous (CL+O): joint capsule and intrinsic ligaments preserved; all muscles, tendons, and fascia removed.
4. Osteology‐only (O): capsule and ligaments removed; bone‐on‐bone interaction guided by articular surface congruence, allowing rolling and sliding without interpenetration and without forcing maximal displacement through unrestricted rotations or translations. Articular cartilage was absent in this condition because measurements were performed on prepared dry bones.

**FIGURE 1:**
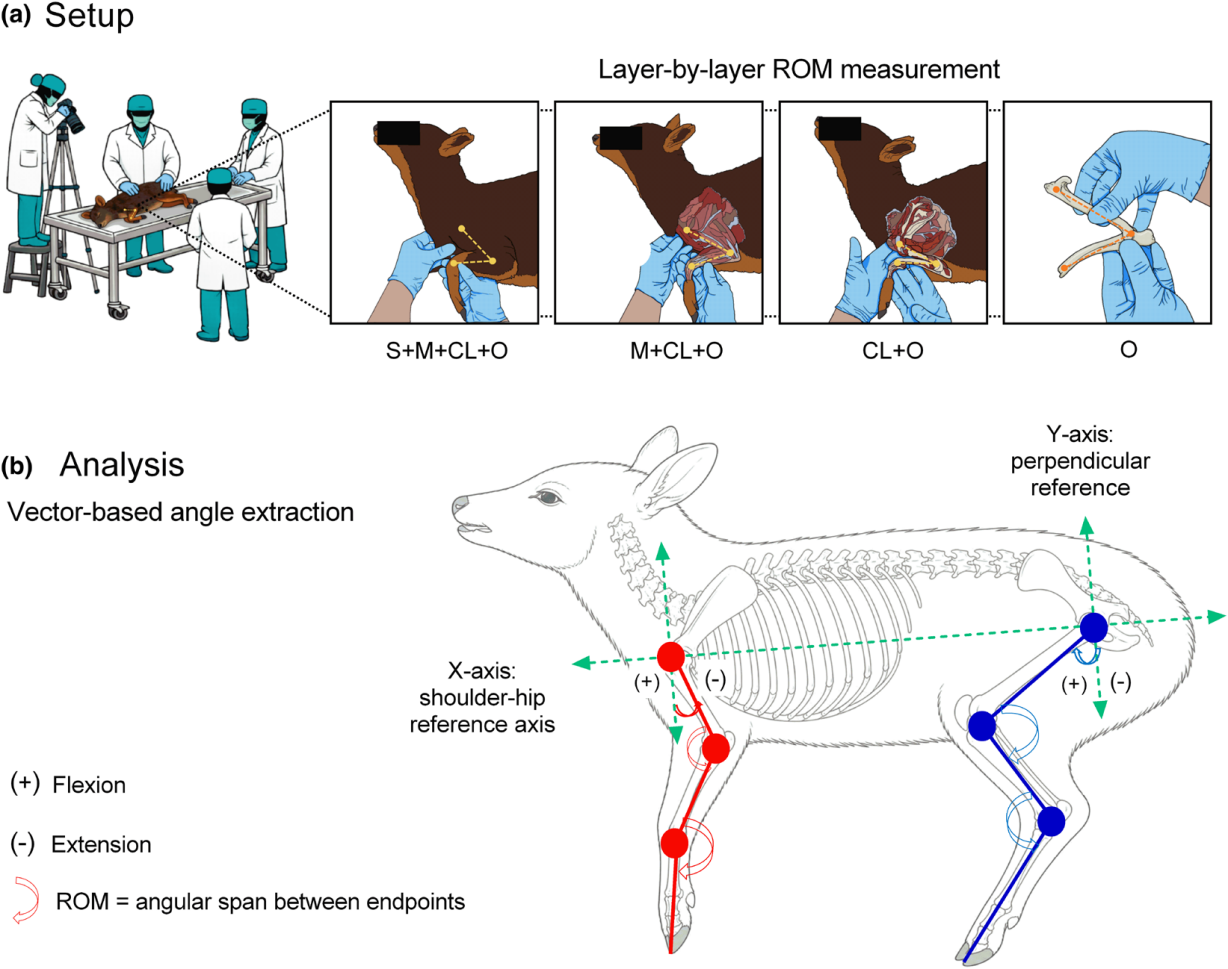
Overview of the experimental workflow for layer‐resolved ex vivo joint range of motion (ROM) and vector‐based angle extraction. (a) Experimental setup and stepwise dissection protocol used to quantify passive flexion–extension‐dominant ROM under four tissue‐layer conditions: S+M+CL+O (skin and subcutaneous tissues, myofascia, capsulo‐ligamentous tissues, and osteology), M+CL+O (skin removed), CL+O (skin and myofascia removed; capsulo‐ligamentous tissues retained), and O (osteology‐only). For each joint and tissue condition, limbs were manually manipulated to identify maximal flexion and maximal extension endpoints. ROM was calculated as the angular span between passive flexion and extension endpoints. (b) Vector‐based extraction of joint angles from the end‐range positions, illustrated on a side‐lying representative specimen consistent with the experimental position used during passive manipulation; forelimb joints are shown in red and hindlimb joints in blue. Joint centres were defined as functional centroids, and segmental axes were constructed by connecting adjacent centroids. Curved arrows indicate the conventional angular directions assigned to flexion (+) and extension (−). For proximal joints, the dashed green *X*‐axis connects the shoulder and hip centroids, and the perpendicular dashed *Y*‐axis provides the reference for angular displacement. For distal joints, the distal vector follows the functional axis of the autopodium. ROM was therefore interpreted as angular excursion between passive endpoints, rather than as absolute joint pose. Representative handling and joint manipulation are documented in Supplementary Video 1 (chilla fox shoulder under the S+M+CL condition).

The same sequence was applied within each limb so that each joint provided repeated measurements across tissue conditions. This layer‐by‐layer experimental sequence is illustrated in Figure 1a.

### 2.5 Dissection

All dissections were performed by an expert technical anatomist at the Instituto de Farmacología y Morfofisiología Veterinaria (Institute of Pharmacology and Veterinary Morphophysiology), Universidad Austral de Chile. The same anatomist conducted all procedures across specimens to minimise variability in tissue removal and preserve comparability across anatomical conditions.

Skin was incised along tension lines and reflected. Subcutaneous tissues were retained for the intact condition and removed in subsequent conditions. Muscles were detached with minimal traction, and tendons were severed close to their insertions under loupe magnification. For the capsulo‐ligamentous condition, the fibrous joint capsule and intrinsic ligaments were preserved intact. Hydration was maintained by periodic application of 0.9% saline during dissection and measurement for the soft‐tissue conditions (S+M+CL+O, M+CL+O and CL+O). The osteology‐only condition was quantified using osteological preparations from the same individuals, based on congruence and relative motion of the articular surfaces. Detailed layer‐removal procedures and representative images for each condition are provided in Supplementary File 3.

### 2.6 Joints examined and anatomical coordinate systems

We examined the shoulder, elbow, wrist (carpal complex), hip, knee (stifle), and ankle (hock; tarsal complex). The protocol targeted flexion–extension‐dominant motion within a sagittal‐oriented movement plane, while recognising that small coupled rotations or translations may occur during passive manipulation.

Joint coordinate systems were defined using functional centroids and segmental axes rather than fixed osteological landmarks. Because osteological landmarks were not consistently visible across tissue conditions, especially in intact specimens, joint centres were identified from passive exploratory movements as approximate functional centroids, based on the apparent centre of rotation observed during mobilisation.

Segmental axes were then defined as lines connecting adjacent functional centroids (e.g. shoulder–elbow, elbow–wrist), representing functional segment orientation rather than strict osteological long axes. For proximal joints (shoulder and hip), the scapula and pelvis were not used as formal reference segments because their position could not be standardised consistently across tissue conditions. Instead, a body‐referenced *X*‐axis was established between the shoulder and hip centroids, and a perpendicular *Y*‐axis was used to quantify angular displacement. This operational reference system was considered appropriate because the primary outcome was total ROM, not absolute joint pose. The centroid‐based coordinate system and vector‐based angle extraction are illustrated in Figure 1b.

Stabilisation and handling points were standardised for each joint to reduce motion transmitted from adjacent segments during passive manipulation.

### 2.7 Passive manipulation and end‐feel definition

End range was reached by manual passive manipulation guided by arthrokinematic principles used in physiotherapy (Kaltenborn, 2004). In this sense, arthrokinematic principles refer to accessory joint motions between articular surfaces, including rolling, sliding (gliding), and spin, which were used qualitatively to maintain articular congruence during passive mobilisation.

Three trained evaluators performed the passive joint manipulations to establish maximal flexion and extension endpoints. Sweep speed was not controlled by metronome; instead, evaluators advanced at a steady pace guided by tissue resistance. Terminal positions were defined at the first firm capsular end feel, operationalised as a consistent rise in resistance without tissue tearing, audible crepitus, or visible articular incongruency, and/or loss of articular congruence in osteology‐only conditions.

If any trial showed tearing, sudden loss of resistance or gross incongruency, that trial was excluded and the endpoint was re‐established on a subsequent evaluation when feasible.

### 2.8 Image capture, landmarking, and digital angle measurement

For each joint and tissue condition, still images were captured at the terminal positions of flexion and extension using a digital camera (Canon EOS Rebel T3) mounted on a rigid tripod. Passive ROM manipulation was performed by three trained evaluators. During image acquisition, photographs and videos were recorded by an author who was not performing the passive manipulation during the recorded trial. All images were digitally recorded and analysed by a single experienced assessor using vector‐based angle measurements to ensure consistency in landmark placement and angle extraction. The overall workflow, including the layer‐by‐layer ROM protocol and the vector‐based angle‐extraction procedure, is summarised in Figure 1.

The digital camera was aligned orthogonally to the intended plane of motion to minimise parallax. A scale bar and a joint‐centre fiducial were included in the field of view. The workflow from ex vivo manipulation to digital angle extraction, and joint‐specific landmark locations, are summarised in Figure 1. Images were analysed in Tracker (v6.1.6; Open‐Source Physics; physlets.org/tracker) using its angle tool. Two reference lines were defined from osteological landmarks representing the proximal and distal segment axes in the joint coordinate system. All landmarks were placed by a single experienced assessor to minimise digitising variability.

Flexion–extension ROM (°) was calculated as the absolute angular difference between maximal flexion and maximal extension, representing the total angular excursion achieved along the flexion–extension‐dominant trajectory.

### 2.9 Data processing and descriptive outcomes

This study reports descriptive outcomes (absolute ROM and percentage change across tissue conditions). Because each taxon was represented by a single specimen, no statistical inference was performed. The study is therefore descriptive and exploratory, focusing on anatomical patterns rather than population‐level inference.

For each specimen and joint, ROM by tissue condition is reported as mean ± SD (CV%) across evaluators (*n* = 3). Layer effects were quantified as (i) absolute differences (°) and (ii) percentage change between successive tissue conditions. Raw measurements and specimen metadata are provided in Supplementary File 1.

Data processing, summary statistics and graphics were produced using GraphPad Prism 6 (GraphPad Software) and IBM SPSS Statistics 20 (IBM Corp.). Digital angle measurements were performed in Tracker v6.1.6.

### 2.10 Use of AI tools

ChatGPT (OpenAI) was used solely to support language editing and improve clarity and readability of the manuscript. No AI tool was used to generate or modify primary data, perform statistical analyses, or produce results. All content was reviewed and approved by the authors, who take full responsibility for the scientific integrity of the work.

## 3 RESULTS

### 3.1 Specimen metadata and handling conditions

Specimen identity, sampling and handling conditions are summarised in Table 1. The four mammals spanned differing postural categories (plantigrade, digitigrade and unguligrade) and body mass classes, providing biological context for interpreting layer‐resolved passive ROM patterns.

### 3.2 Overview of layer‐resolved passive ROM patterns

**FIGURE 2:**
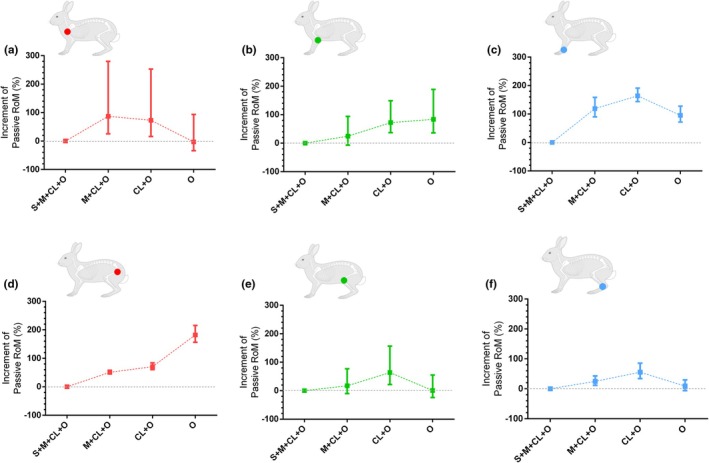
Layer‐resolved percentage change in passive flexion–extension‐dominant range of motion (ROM) for the rabbit (*Oryctolagus cuniculus*), expressed relative to the intact baseline condition (S+M+CL+O; set to 0%). Forelimb joints are shown in (a) shoulder, (b) elbow and (c) wrist; hindlimb joints are shown in (d) hip, (e) knee and (f) ankle. Points represent the mean across three evaluators (*n* = 3) for each tissue condition, and error bars indicate ± SD. The horizontal grey line marks the intact baseline. Grey anatomical insets identify the joint represented in each panel, with the coloured centroid corresponding to the plotted joint. Tissue‐layer abbreviations follow Figure 1.

**FIGURE 3:**
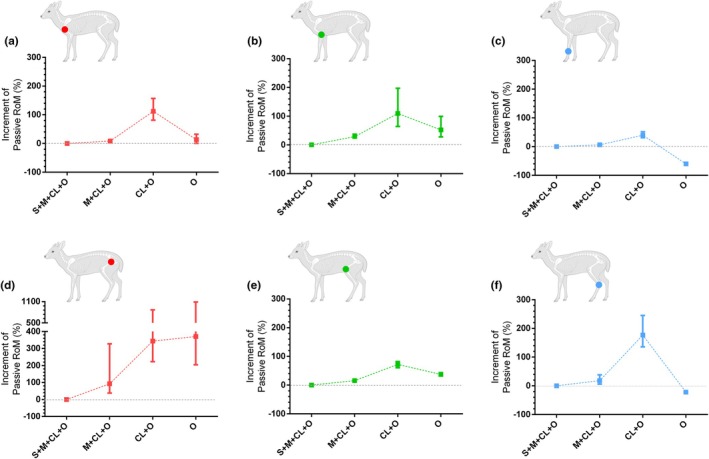
Layer‐resolved percentage change in passive flexion–extension‐dominant range of motion (ROM) for the pudu (*Pudu puda*), expressed relative to the intact baseline condition (S+M+CL+O; set to 0%). Forelimb joints are shown in (a) shoulder, (b) elbow and (c) wrist; hindlimb joints are shown in (d) hip, (e) knee and (f) ankle. Points represent the mean across three evaluators (*n* = 3) for each tissue condition, and error bars indicate ± SD. The horizontal grey line marks the intact baseline. Grey anatomical insets identify the joint represented in each panel, with the coloured centroid corresponding to the plotted joint. Tissue‐layer abbreviations follow Figure 1.

**FIGURE 4:**
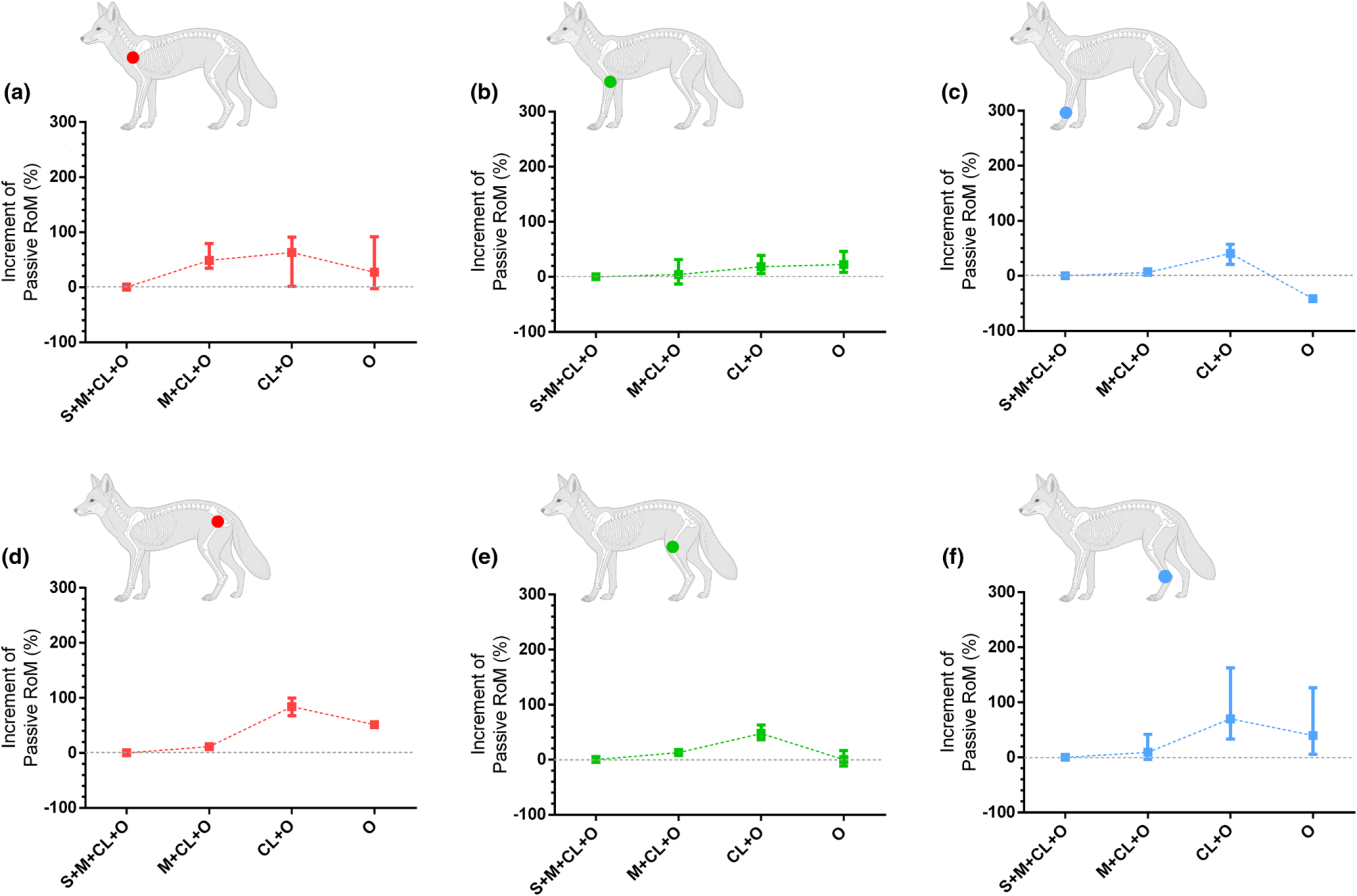
Layer‐resolved percentage change in passive flexion–extension‐dominant range of motion (ROM) for the chilla fox (*Lycalopex griseus*), expressed relative to the intact baseline condition (S+M+CL+O; set to 0%). Forelimb joints are shown in (a) shoulder, (b) elbow and (c) wrist; hindlimb joints are shown in (d) hip, (e) knee and (f) ankle. Points represent the mean across three evaluators (*n* = 3) for each tissue condition, and error bars indicate ± SD. The horizontal grey line marks the intact baseline. Grey anatomical insets identify the joint represented in each panel, with the coloured centroid corresponding to the plotted joint. Tissue‐layer abbreviations follow Figure 1.

**FIGURE 5:**
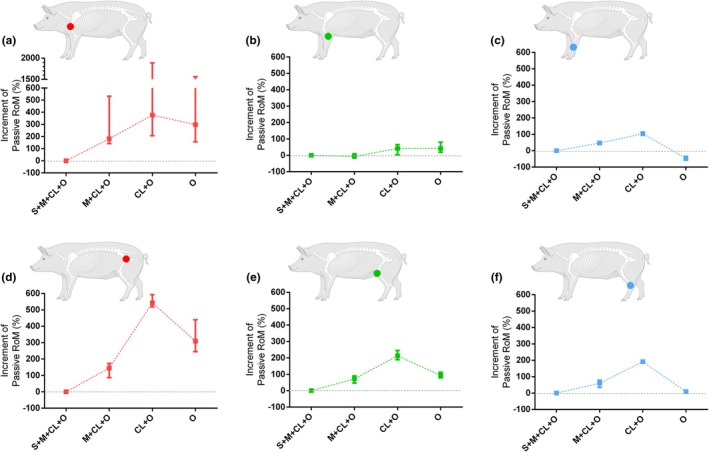
Layer‐resolved percentage change in passive flexion–extension‐dominant range of motion (ROM) for the pig (*Sus scrofa domestica*), expressed relative to the intact baseline condition (S+M+CL+O; set to 0%). Forelimb joints are shown in (a) shoulder, (b) elbow and (c) wrist; hindlimb joints are shown in (d) hip, (e) knee and (f) ankle. Points represent the mean across three evaluators (*n* = 3) for each tissue condition, and error bars indicate ± SD. The horizontal grey line marks the intact baseline. Grey anatomical insets identify the joint represented in each panel, with the coloured centroid corresponding to the plotted joint. Tissue‐layer abbreviations follow Figure 1.

**FIGURE 6:**
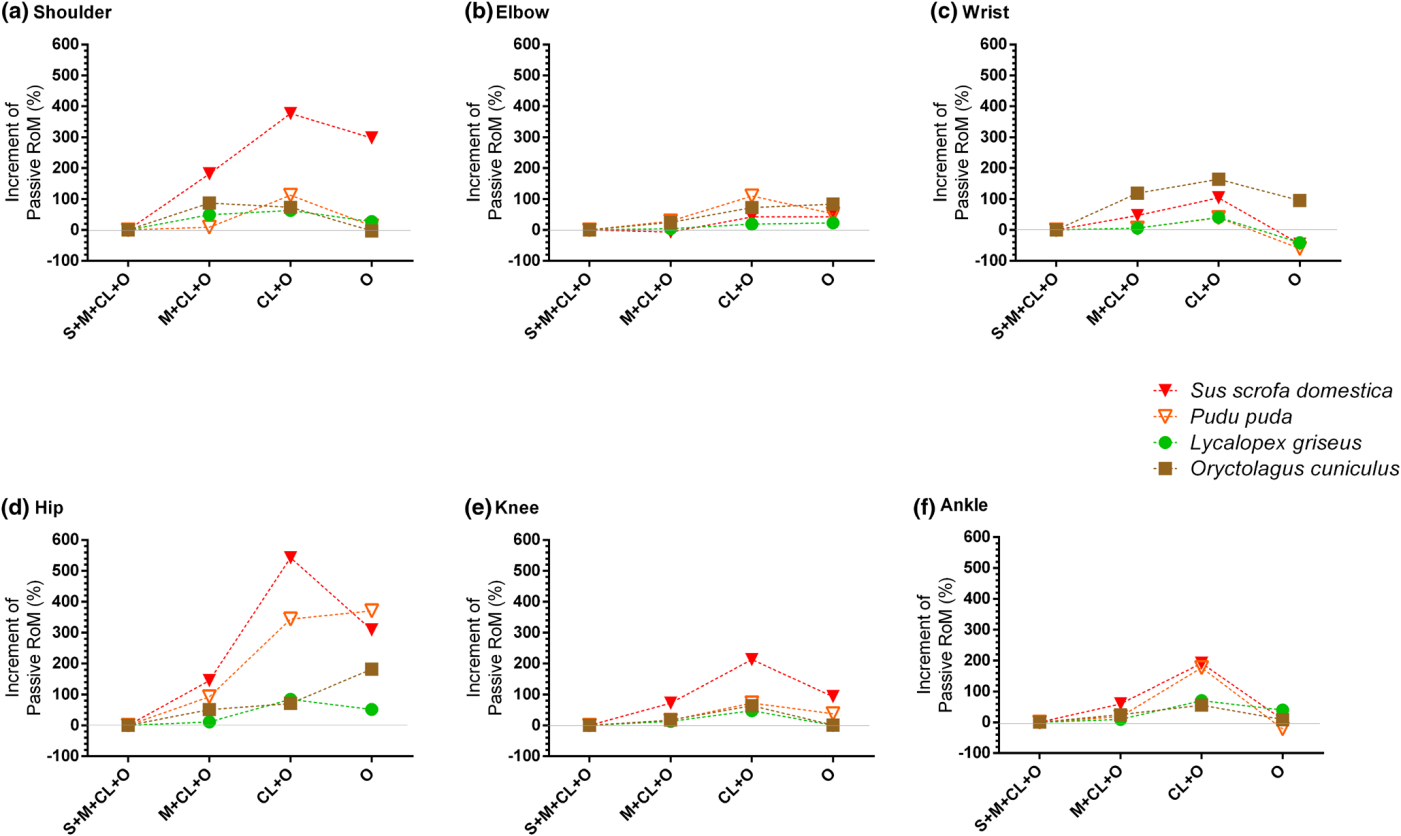
Cross‐taxon comparison of layer‐resolved changes in passive flexion–extension‐dominant ROM across limb joints. Percentage change in passive flexion–extension‐dominant range of motion (ROM) relative to the intact baseline condition (S+M+CL+O; set to 0%) for four mammals, plotted across successive tissue conditions for each joint: (a) shoulder, (b) elbow, (c) wrist, (d) hip, (e) knee and (f) ankle. Values are expressed as the increment of passive ROM (%) relative to baseline (0%), derived from the absolute ROM reported in Table 2. The horizontal grey line marks the intact baseline. A common *y*‐axis scale was retained across panels to allow direct comparison of the magnitude of layer‐resolved ROM changes among joints. Lines and symbols denote species: *Sus scrofa domestica* (red triangles), *Pudu puda* (orange open triangles), *Lycalopex griseus* (green circles) and *Oryctolagus cuniculus* (brown squares). Tissue conditions are: S, skin and subcutaneous tissue; M, myofascia; CL, capsulo‐ligamentous tissues; O, osteology‐only. ROM values represent means across three evaluators (*n* = 3 per joint and tissue condition). Dispersion around species‐specific means is shown separately in Figures 2, 3, 4, 5 and reported in Table 2.

Across all taxa and joints, passive flexion–extension‐dominant ROM varied with tissue condition in a joint‐ and species‐specific manner (Table 2; Figures 2, 3, 4, 5, 6). Absolute ROM values are reported in Table 2 (mean ± SD; CV%), whereas Figures 2, 3, 4, 5, 6 show the corresponding percentage changes relative to the intact baseline (S+M+CL+O). In most joints, ROM increased from the intact condition to intermediate tissue conditions, but osteology‐only measurements did not consistently produce the highest ROM. Several joints showed non‐monotonic sequences in which ROM peaked at CL+O and then decreased in the osteology‐only condition (O), most prominently at the wrist and, in some taxa, at the ankle and knee (Figures 3, 4, 5, 6). Measurement dispersion across evaluators varied among joints and tissue conditions (Table 2), with CV values ranging from 0.0% to 32.1%. The lowest dispersion was observed in the rabbit wrist under the M+CL+O condition (129.3 ± 0.0°; CV = 0.0%), whereas the highest dispersion occurred in the pig shoulder under the intact S+M+CL+O condition (18.4 ± 5.9°; CV = 32.1%). Other relatively high CV values were observed in the pudu hip under the intact S+M+CL+O condition (25.2%), the chilla fox shoulder under CL+O (24.5%) and the pig shoulder under M+CL+O (22.0%). By contrast, several intermediate and osteology‐only conditions showed low dispersion, often below 5%, including rabbit shoulder under CL+O (0.6%), rabbit ankle under CL+O (0.41%) and pig wrist under CL+O (0.9%). Overall, higher dispersion tended to occur in some intact‐condition measurements and in joints that were more complex to manipulate consistently, whereas several intermediate conditions showed highly repeatable passive endpoints.

**TABLE 2:** Passive flexion–extension range of motion (ROM, °) by tissue condition and joint in four mammals.

Species	Tissue condition	Shoulder	Elbow	Wrist	Hip	Knee	Ankle
*Oryctolagus cuniculus*	S+M+CL+O	73.8 ± 15.3 (20.8%)	72.8 ± 11.0 (15.2%)	59.0 ± 3.6 (6.1%)	71.4 ± 3.5 (4.9%)	95.0 ± 14.4 (15.2%)	102.2 ± 7.0 (6.9%)
	M+CL+O	138 ± 1 (0.7%)	90.7 ± 1.0 (1.1%)	129.3 ± 0.0 (0.0%)	107.5 ± 3.5 (3.3%)	111.2 ± 2.6 (2.3%)	127.5 ± 2.5 (2.0%)
	CL+O	127.9 ± 0.8 (0.6%)	125.9 ± 4.9 (3.9%)	155.7 ± 4.0 (2.6%)	122.0 ± 2.7 (2.2%)	155.5 ± 1.5 (1.0%)	158.9 ± 0.7 (0.41%)
	O	72 ± 1.0 (1.5%)	133.7 ± 1.1 (0.8%)	115.3 ± 0.6 (0.5%)	201.5 ± 1.5 (0.8%)	95.8 ± 1.6 (1.6%)	111.4 ± 0.5 (0.5%)
*Pudu puda*	S+M+CL+O	64.8 ± 4.8 (7.4%)	61.3 ± 7.8 (12.8%)	128.4 ± 6.4 (5.0%)	25.6 ± 6.4 (25.2%)	82.6 ± 3.2 (3.8%)	58.6 ± 5.8 (9.9%)
	M+CL+O	70.3 ± 4.8 (6.9%)	79.0 ± 8.7 (11.8%)	136.1 ± 8.2 (6.0%)	49.3 ± 3.3 (6.7%)	95.4 ± 5.2 (5.5%)	69.3 ± 3.3 (4.8%)
	CL+O	137.5 ± 0.7 (0.5%)	128.3 ± 1.7 (1.3%)	179.6 ± 3.6 (2.0%)	113.7 ± 8.4 (7.4%)	142.3 ± 9.0 (6.3%)	162.2 ± 4.0 (2.5%)
	O	73.6 ± 1.5 (2.0%)	93.3 ± 4.0 (4.3%)	51.1 ± 0.9 (1.8%)	120.6 ± 2.5 (2.1%)	113.3 ± 2.5 (2.2%)	45.9 ± 4.0 (8.7%)
*Lycalopex griseus*	S+M+CL+O	78.1 ± 11.7 (15.0%)	109.5 ± 10.2 (9.3%)	106.1 ± 3.8 (3.6%)	88.1 ± 1.0 (1.1%)	82.6 ± 3.2 (3.8%)	77.9 ± 13.6 (17.4%)
	M+CL+O	116.2 ± 11.4 (9.8%)	114.1 ± 1.4 (1.2%)	112.5 ± 1.5 (1.3%)	98.1 ± 2.2 (2.2%)	123.5 ± 10.2 (8.3%)	85.1 ± 9.0 (10.6%)
	CL+O	127.5 ± 31.2 (24.5%)	129.9 ± 5.3 (4.0%)	149.2 ± 13.1 (8.8%)	162.2 ± 7.4 (4.6%)	161.4 ± 5.8 (3.6%)	132.5 ± 6.6 (5.0%)
	O	99.3 ± 2.1 (2.2%)	134.2 ± 4.5 (3.4%)	62.2 ± 4.0 (6.5%)	133.4 ± 1.4 (1.1%)	109.8 ± 2.0 (1.8%)	108.9 ± 3.6 (3.3%)
*Sus scrofa domestica*	S+M+CL+O	18.4 ± 5.9 (32.1%)	53.5 ± 5.1 (9.5%)	76.6 ± 2.1 (2.8%)	21.0 ± 2.9 (13.8%)	82.6 ± 3.2 (3.8%)	40.6 ± 2.1 (5.1%)
	M+CL+O	51.7 ± 11.4 (22.0%)	50.0 ± 2.0 (4.1%)	112.4 ± 4.8 (2.2%)	51.3 ± 10.3 (20.1%)	61.9 ± 6.8 (11.1%)	64.7 ± 6.8 (10.6%)
	CL+O	87.6 ± 5.5 (6.3%)	75.9 ± 13.5 (17.8%)	156.2 ± 1.5 (0.9%)	134.7 ± 15.8 (11.7%)	112.9 ± 2.9 (2.5%)	118.3 ± 6.1 (5.2%)
	O	73.1 ± 4.6 (6.2%)	76.1 ± 1.0 (1.3%)	41.6 ± 4.7 (11.2%)	85.7 ± 4.5 (5.3%)	69.5 ± 1.8 (2.6%)	44.3 ± 3.3 (7.4%)

*Note*: Values are passive flexion–extension ROM (°) reported as mean ± SD (CV%) across three evaluators (*n* = 3) for each joint and tissue condition. S, skin and subcutaneous tissue; M, myofascia; CL, capsulo‐ligamentous tissues; O, osteology‐only. Representative calibrated images illustrating the dissection sequence and digital angle extraction are provided in Supplementary File 3.

### 3.3 Species‐specific layer‐resolved passive ROM patterns

Rabbit (*O. cuniculus*) (Table 2; Figure 2) forelimb joints showed pronounced increases following removal of skin and subcutaneous tissues (M+CL+O), most notably at the shoulder, which rose from 73.8° (S+M+CL+O) to 138.0° (M+CL+O) (Figure 2a). Elbow ROM increased progressively across layers, reaching its maximum in the osteology‐only condition (133.7°) (Figure 2b). Wrist ROM increased to a maximum at CL+O (155.7°) and then decreased in the osteology‐only condition (115.3°) (Figure 2c). In the hindlimb, hip ROM exhibited the largest net increase in absolute ROM, rising from 71.4° (S+M+CL+O) to 201.5° (O) (Figure 2d). Knee ROM increased to a maximum at CL+O (155.5°) and returned towards intact values in the osteology‐only condition (95.8°) (Figure 2e). The ankle increased to 158.9° (CL+O) and then decreased to 111.4° (O), yielding a modest net increase relative to the intact condition (Figure 2f).

Pudu deer (*P. puda*) (Table 2; Figure 3) showed pronounced joint‐specific responses. In the forelimb, shoulder ROM increased to a maximum at CL+O (137.5°) but declined thereafter, with the osteology‐only condition (73.6°) close to the intact value (64.8°) (Figure 3a). Elbow ROM increased to 128.3° (CL+O) and then decreased to 93.3° (O) (Figure 3b). The wrist showed a marked non‐monotonic pattern, peaking at CL+O (179.6°) but dropping sharply to 51.1° in the osteology‐only condition, which was below the intact value (128.4°) (Figure 3c). In the hindlimb, hip ROM showed the strongest net increase in absolute ROM, rising from 25.6° (S+M+CL+O) to 120.6° (O) (Figure 3d). Knee ROM also showed an intermediate‐condition maximum, increasing from 82.6° (S+M+CL+O) to 142.3° (CL+O), before decreasing to 113.3° in the osteology‐only condition (Figure 3e). The ankle exhibited a similar reversal, peaking at CL+O (162.2°) and decreasing to 45.9° (O), again below the intact value (58.6°) (Figure 3f). These reversals are also apparent in the cross‐taxon comparison (Figure 6), where pudu deer shows among the largest negative final‐step changes (CL+O to O) at the wrist and ankle.

Chilla fox (*L. griseus*) (Table 2; Figure 4) displayed intermediate‐condition maxima at multiple joints. In the forelimb, shoulder ROM increased from 78.1° (S+M+CL+O) to a maximum at CL+O (127.5°), followed by a decrease to 99.3° (O) (Figure 4a). Elbow ROM increased more steadily across layers, reaching its maximum in the osteology‐only condition (134.2°) (Figure 4b). Wrist ROM again showed a strong non‐monotonic response, increasing to 149.2° (CL+O) and then decreasing to 62.2° (O), well below the intact value (106.1°) (Figure 4c). In the hindlimb, hip ROM rose to 162.2° at CL+O and then decreased to 133.4° (O) (Figure 4d). Knee ROM increased to 161.4° (CL+O) and decreased to 109.8° (O) (Figure 4e). The ankle increased to 132.5° (CL+O) and decreased to 108.9° (O), remaining above the intact value (77.9°) (Figure 4f). Overall, the wrist showed the clearest reduction in osteology‐only ROM (Figures 4 and 6).

Pig (*S. scrofa domestica*) (Table 2; Figure 5) exhibited the largest proportional increases across layers in several joints, accompanied by large absolute shifts. In the forelimb, shoulder ROM increased from 18.4° (S+M+CL+O) to 87.6° (CL+O) and then decreased to 73.1° (O) (Figure 5a). Elbow ROM increased to 76.1° (O) (Figure 5b). Wrist ROM showed a pronounced osteology‐only reduction: after peaking at 156.2° (CL+O), it fell to 41.6° (O), below the intact value (76.6°) (Figure 5c). In the hindlimb, hip ROM increased from 21.0° (S+M+CL+O) to 134.7° (CL+O) and decreased to 85.7° (O) (Figure 5d). Knee ROM increased to 112.9° (CL+O) but decreased to 69.5° (O), also below the intact value (82.6°) (Figure 5e). Ankle ROM peaked at 118.3° (CL+O) and decreased to 44.3° (O), remaining close to the intact value (40.6°) (Figure 5f). Collectively, these trajectories indicate that the largest expansions occurred at intermediate tissue conditions, whereas osteology‐only values could be reduced in distal and multi‐element joints (Figures 5 and 6).

### 3.4 Cross‐species comparison by joint

Across taxa, the shoulder tended to show substantial increases across layers, although the condition producing maximum ROM varied among taxa (M+CL+O in rabbit versus CL+O in pudu deer, chilla fox, and pig; Figures 2, 3, 4, 5, 6a; Table 2). The elbow generally exhibited more progressive increases and smaller reversals than distal joints, with osteology‐only values often remaining close to or above intermediate‐condition values (Figures 2, 3, 4, 5, 6b). In contrast, the wrist showed the most frequent and pronounced non‐monotonicity, with ROM commonly peaking at CL+O and then decreasing sharply in the osteology‐only condition in three of four taxa (pudu deer, chilla fox, and pig), and decreasing more moderately in rabbit (Figures 2, 3, 4, 5, 6c; Table 2). In the hindlimb, the hip showed consistently large gains from the intact condition to later tissue conditions, with particularly strong net increases in rabbit and pudu deer (Figures 2, 3, 4, 5, 6d; Table 2). The knee displayed mixed behaviour across species, often increasing to intermediate maxima but showing variable final‐step changes to osteology‐only values (Figures 2, 3, 4, 5, 6e). The ankle also showed joint‐ and taxon‐specific trajectories, with intermediate‐condition maxima followed by variable reductions in the osteology‐only condition, most notably in pudu deer and pig (Figures 2, 3, 4, 5, 6f).

## 4 DISCUSSION

**FIGURE 7:**
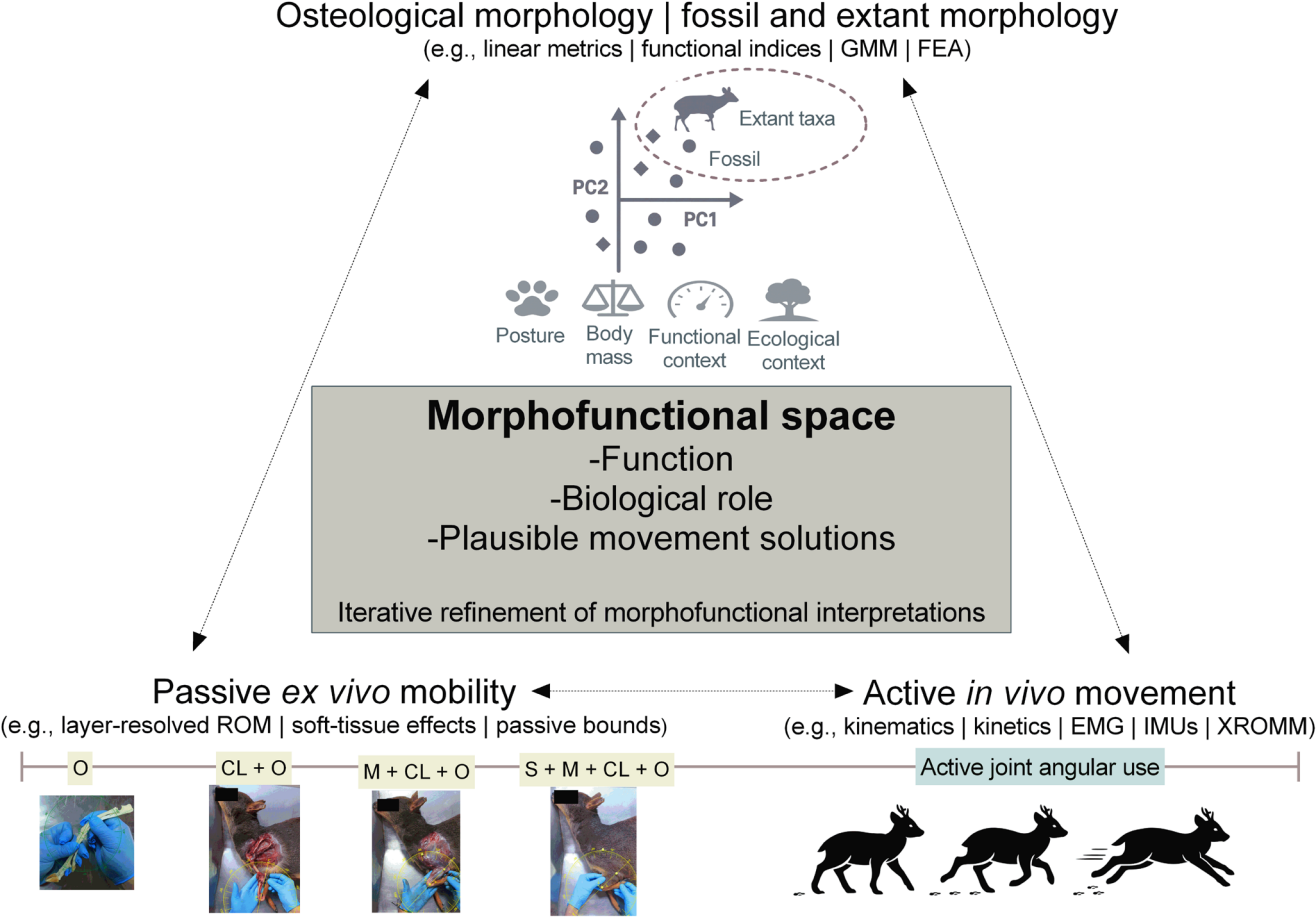
Morphofunctional space as an integrative framework linking osteological morphology, passive ex vivo mobility and active in vivo movement. In this framework, the present study contributes primarily to the passive ex vivo mobility stream by quantifying layer‐resolved soft‐tissue effects on flexion–extension‐dominant ROM in extant mammals. Osteological morphology includes fossil and extant morphology and may be quantified using linear metrics, functional indices, geometric morphometrics (GMM) and finite element analysis (FEA). Passive ex vivo mobility provides tissue‐informed evidence on how anatomical layers and soft‐tissue effects modify the passive mobility envelope, including layer‐resolved ROM and passive bounds. Active in vivo movement provides behavioural evidence on how part of this potential envelope is selected and coordinated during locomotor or functional tasks, through kinematics, kinetics, electromyography, inertial measurement units and XROMM‐based approaches. These evidence streams are connected through reciprocal calibration rather than a one‐way sequence, allowing iterative refinement of morphofunctional interpretations within a morphofunctional space. Biological descriptors, such as posture, body mass, functional context and ecological context, provide contextual grouping variables for analogue‐informed interpretation rather than direct predictors of ROM or criteria for single‐analogue selection. Ultimately, this framework may support future palaeobiological applications by helping to evaluate plausible functional interpretations in extinct taxa through cautious comparison with extant anatomical and movement data.

This study provides a layer‐resolved, ex vivo assessment of passive flexion–extension ROM across six major limb joints in four extant mammals spanning differing postural categories. The principal result is that tissue removal does not yield a uniform or consistently progressive expansion of joint ROM. Instead, responses were joint‐ and taxon‐specific, with frequent intermediate‐condition maxima and, in several distal joints, reduced apparent ROM following removal of the capsulo‐ligamentous envelope. These outcomes reinforce that osteological geometry alone is an incomplete proxy for functional joint mobility, because the soft‐tissue envelope can both constrain motion and stabilise joint alignment in ways that shape the realised passive movement space (Arnold et al., 2014; Broyde et al., 2021; Hutson & Hutson, 2012). This conceptual point is developed further through a conceptual inverse framework that integrates osteology‐only estimates, layer‐resolved passive envelopes and in vivo joint use (Figure 7) and complements broader biomechanical inference approaches in vertebrate palaeontology, including recent multi‐degree‐of‐freedom and pose‐space approaches (Manafzadeh et al., 2024; Manafzadeh & Gatesy, 2021), that necessarily incorporate soft‐tissue assumptions when reconstructing function from hard‐tissue evidence (Brassey et al., 2017).

### 4.1 Soft tissues as constraints and as stabilisers of joint motion

In mechanistic terms, these results indicate that different tissue layers can shift apparent ROM via two non‐exclusive roles: constraining excursion and stabilising alignment. More broadly, these tissues act as modulators of joint motion by influencing articular congruence, joint‐centre stability and the trajectory of movement. This helps explain why osteology‐only values were not consistently maximal, particularly in distal, multi‐element complexes.

Across taxa, several joints increased markedly from the intact condition to intermediate tissue conditions, indicating substantial constraint imposed by superficial layers and myofascial structures in some anatomical contexts. However, the osteology‐only condition did not consistently yield the highest ROM. In the wrist of the pudu deer, chilla fox, and pig, ROM peaked at CL+O and then dropped sharply in O, in some cases falling below intact values. Similar reversals were evident in the pudu ankle and pig knee. A parsimonious interpretation is that capsulo‐ligamentous tissues are not merely passive restraints; they also provide joint‐centred guidance, limit translations and help maintain congruency across multi‐element complexes. When these tissues are removed, altered joint spacing and alignment can shift effective centres of rotation and promote earlier impingement or incongruent contact, reducing the apparent flexion–extension arc. This pattern is consistent with recent anatomical and modelling work showing that internal joint distance, joint spacing and articular congruence strongly influence reconstructed mobility envelopes and should be considered explicitly when defining articular contact in functional reconstructions (Demuth et al., 2025; Lee et al., 2023; Scheidt et al., 2026). This interpretation is consistent with evidence that joint spacing, used as a proxy for articular cartilage thickness, can influence reconstructed mobility envelopes even when osteological form remains constant (Wiseman et al., 2022). Furthermore, it aligns with recent work demonstrating that joint mobility cannot be fully understood without considering coupled rotations and translations across multiple degrees of freedom (Brocklehurst et al., 2025; Manafzadeh & Gatesy, 2021). In this sense, osteology‐only ROM can represent a mechanically altered state rather than an upper bound on biologically feasible joint motion.

The observation that distal, multi‐element joints were especially prone to non‐progressive patterns is also anatomically intuitive. In the wrist and ankle, multiple articular surfaces and coupled motions mean that passive flexion–extension is highly sensitive to small changes in alignment, tensioning pathways, and contact location. Removing intrinsic constraints can therefore reduce, rather than increase, the flexion–extension arc if the joint settles into a less favourable alignment for that particular axis of motion. This interpretation is consistent with recent ex vivo and XROMM‐based studies showing that multi‐element joints are particularly sensitive to subtle variations in alignment and articular spacing (Demuth et al., 2025). This supports the view that soft tissues should not be treated as a single uniform modifier; their influence depends on which anatomical layer is considered and whether that layer predominantly contributes restraint, guidance, congruency or elastic energy storage under load (Alexander, 2002; Burgio et al., 2023).

### 4.2 Measurement consistency as an anatomical signal, not only a methodological limitation

Variation among evaluators, summarised by SD and CV%, was not constant across joints or tissue conditions (Table 2). Dispersion tended to be higher in several intact measurements and in joints that were more complex to manipulate consistently, whereas multiple intermediate tissue conditions showed comparatively low CV values. This pattern is consistent with the idea that, when multiple soft‐tissue layers are present, passive end range reflects distributed resistance from skin, fascia, muscle and periarticular tissues, producing a more gradual and potentially evaluator‐dependent endpoint.

This observation reinforces that variability is not solely methodological noise, but may reflect underlying anatomical and mechanical ambiguity in passive endpoints. In particular, when several tissue layers contribute simultaneously to resistance, the transition between compliant and restrictive behaviour becomes less discrete, leading to broader and less sharply defined end‐feel boundaries.

By contrast, after removal of selected layers, the endpoint may become sharper and more repeatable, for example, a clearer capsular end feel or an earlier osteological stop, improving between‐evaluator consistency. Accordingly, CV patterns are best interpreted as joint‐ and condition‐specific indicators of endpoint sharpness, rather than as a single quality threshold applied uniformly across the protocol.

Importantly, this study intentionally used a clinically grounded, manual arthrokinematic approach to define end feel, rather than a force‐controlled mechanical rig. Evaluators were trained to apply arthrokinematic principles and a shared passive end‐feel criterion, which likely contributed to the generally low between‐evaluator dispersion observed across many joints and tissue conditions, with particularly small CV values in several intermediate and osteology‐only measurements (Table 2). Although strict cross‐study comparisons are limited by differences in taxa, joint structure, and experimental context, this overall pattern is consistent with the view that explicit training, standardised handling, and a shared endpoint definition can improve measurement consistency. Comparable EPB repeated‐measures ROM studies show that between‐observer effects can be minimised when handling and endpoint criteria are standardised, while absolute angles remain sensitive to endpoint definition, tissue condition and the configuration of adjacent or coupled joints (Hutson & Hutson, 2012, 2013, 2014; Manafzadeh, 2020). Rather than treating dispersion purely as noise, it may therefore be informative about which joints and tissue conditions yield the most ambiguous passive endpoint, particularly in distal complexes where small translations and coupled rotations can occur. The present results extend these cautions to mammals and suggest that dispersion itself may scale with joint complexity and with the number of intact layers contributing to the passive resistance profile.

Thus, the reliability component of this study should be interpreted not only as quality control, but also as an anatomical indicator of how clearly different tissue configurations define passive end range.

### 4.3 Interpreting patterns in relation to posture and biological context

Although this dataset was not designed for inferential comparisons among species (*n* = 1 per taxon), the layer‐resolved trajectories provide descriptive contrasts that can be interpreted in relation to posture and the habitual loading regimes associated with different limb designs.

Because postural and locomotor categories behave as continua rather than discrete states (Carrano, 1999; Gambaryan, 1974), they are used here as comparative anatomical descriptors rather than deterministic predictors of ROM.

The plantigrade rabbit showed large increases at the shoulder and hip across the dissection sequence, consistent with substantial constraint imposed by superficial and myofascial layers at proximal joints. By contrast, the digitigrade chilla fox more often exhibited intermediate‐condition maxima and a pronounced osteology‐only reduction at the wrist, suggesting that distal joint mobility may depend strongly on periarticular guidance and joint congruency rather than on osteological geometry alone. The unguligrade taxa showed distinct distal joint behaviour: the pudu deer displayed marked osteology‐only reductions at both wrist and ankle, whereas the pig combined large proportional expansions at intermediate conditions with osteology‐only reductions at the wrist and knee.

These descriptive differences are compatible with the premise that posture, segment proportions, and habitual loading shape soft‐tissue architecture and material properties. In mammals, tendons, ligaments, and fascial structures can vary widely in stiffness and modulus, with large differences even among broadly comparable limb designs (Burgio et al., 2023). In addition, mechanosensory systems and tissue regulation are evolutionarily tuned to locomotor mechanics, and scaling and structural safety factors influence how stresses are distributed and stability margins are maintained across body sizes and limb configurations (Aiello et al., 2017; Alexander, 1981; Biewener, 2005). Comparative evidence from soft‐tissue anatomy also supports a functional gradient along the limb: hindlimb muscle architecture in small‐bodied generalist mammals is broadly conserved, yet differences tend to emerge towards the distal limb, consistent with the expectation that structures closer to the substrate and to multi‐element joint complexes are more likely to reflect task‐specific stabilisation and control demands (Wright et al., 2022). Under this framework, similar osteological configurations may yield different passive envelopes depending on the composition, organisation and mechanical role of the surrounding soft tissues (Broyde et al., 2021).

More broadly, this interpretative approach follows an inverse logic widely used in palaeobiology, in which preserved structure is linked to dynamic function by integrating extant experiments with constrained modelling and comparative datasets from living analogues. This logic is consistent with recent syntheses of palaeobiological reconstruction as an explicitly testable chain of inference, in which fossil observations are interpreted through comparative and experimental toolkits derived from extant systems, while each inferential step remains open to refinement or falsification (Benton & Rayfield, 2026). In locomotion, joint angles measured in vivo reflect active, coordinated use of the limb and typically explore only a fraction of the available passive space, varying with task, speed, and posture; moreover, joint angular excursion profiles provide a useful comparative descriptor across cyclical behaviours and functional systems (Catavitello et al., 2018; Fischer & Blickhan, 2006; Granatosky et al., 2019; Medina‐González, 2026). A recent example in mammals shows how osteological geometry can be linked to locomotor function through constrained modelling: a calcaneal lever‐based model predicts stance‐phase ankle angle across terrestrial mammals from skeletal measurements, with close correspondence to in vivo observations (Mizuno & Fujiwara, 2025). Importantly, such reconstructions target a task‐specific operating angle under habitual loading rather than the full passive envelope, and they rely on assumptions about articular alignment and spacing that may not be preserved by osteology‐only manipulations. This distinction aligns with the present results, in which distal multi‐element joints were particularly sensitive to the loss of periarticular guidance across tissue conditions.

From a functional perspective, the contrast between proximal and distal joints may reflect a shift from joints where soft tissues primarily limit gross angular excursion, often proximally, to joints where soft tissues also maintain alignment and congruency within multi‐element or multi‐articular complexes, often distally. In distal joints, posture‐related differences in autopodial orientation and load distribution may amplify small translations and coupled rotations, making the flexion–extension arc especially sensitive to subtle alignment changes across tissue conditions. Tendon and ligament function is not limited to restricting motion; these tissues can store and return energy, guide movement trajectories and stabilise joints under load (Alexander, 2002). Work on ungulate tarsal mechanics further illustrates how mobility can be partitioned across a multi‐element complex in ways that are not captured by a single osteological feature, and how joint congruency and periarticular guidance can be central to expressing functional excursion (Takeda et al., 2026). In addition, changes in internal joint distance, used as a proxy for cartilage thickness, and in translation–rotation interactions can modify the apparent mobility envelope even when osteological form is unchanged (Manafzadeh & Gatesy, 2021; Wiseman et al., 2022). The present ex vivo results therefore provide an anatomical basis for interpreting why some joints show substantial release following superficial dissection, whereas others require intact periarticular constraints to express a larger flexion–extension arc.

### 4.4 Integrating layer‐resolved ROM into morphofunctional inference

Layer‐resolved ROM can contribute to morphofunctional inference because functional interpretation often proceeds in the reverse direction: preserved anatomy provides the osteological template, whereas the mobility envelope in life emerged from that template embedded within a soft‐tissue system. This inverse logic is especially relevant in palaeobiology, where functional reconstructions are built by combining descriptions of preserved material with experimental evidence from extant proxies and, where appropriate, modelling approaches that explore a constrained solution space (Benton & Rayfield, 2026; Nyakatura, 2017). It is also consistent with broader views of the fossil record as a biological laboratory for framing and testing hypotheses about phenotypes and their functions through time, particularly when integrated with experimental and modelling approaches from extant systems (Benton & Rayfield, 2026; Jablonski & Shubin, 2015). Within this context, the present framework treats layer‐resolved ex vivo ROM as one anatomical contribution to a broader chain of inference, rather than as a direct translation from osteological morphology to biological function.

This inverse logic can also be framed through the conceptual role of joint mobility as an intermediate link between anatomical form and biological function. Manafzadeh (2023) argued that joint mobility helps divide the otherwise difficult form–function problem into two more tractable relationships: form–mobility, describing how morphology shapes potential motion, and mobility–function, describing how potential motion relates to observed in vivo movement. The framework proposed here follows this logic but separates three complementary evidence streams: osteological form, layer‐resolved passive ex vivo mobility and active in vivo angular use. This separation is not arbitrary: passive ex vivo mobility captures how periarticular soft tissues constrain, stabilise, or facilitate the mobility envelope around an osteological template, whereas active in vivo angular use reflects how musculoskeletal levers, muscle force directions and neuromotor control select only part of that envelope during behaviour. Thus, function and biological role are refined through reciprocal calibration among evidence streams rather than inferred from morphology alone. This distinction is consistent with Lauder's view that form–function relationships are not necessarily one‐to‐one, because similar forms may support different functions and different forms may converge on similar functions, with neuromotor control acting as an additional source of functional variability (Lauder, 1995). It also aligns with the reverse‐engineering logic of Nyakatura et al. (2019), in which fossil anatomy, trackways, extant in vivo locomotor data, simulations and robotics were integrated to filter plausible locomotor solutions.

Recent work has underscored that reconstructing articular function in extinct taxa requires attention to the full dimensionality of joint motion, including translations and coupled rotations, rather than relying solely on simplified hinge‐like assumptions (Manafzadeh & Gatesy, 2021). Previous palaeobiological ROM studies have also used physical or hybrid physical–virtual manipulation of osteological material to evaluate feasible mobility and functional posture, including manual manipulation of theropod forelimb casts (Senter & Robins, 2005) and simulated ROM analyses of the sauropod pes under bone‐to‐bone and hypothetical cartilage scenarios (Jannel et al., 2019).

Rather than seeking a single extant analogue, recent integrative reconstructions have emphasised the value of combining multiple, partially independent constraints. For example, Nyakatura et al. (2019) reconstructed plausible locomotor solutions for the stem amniote *Orobates pabsti* by integrating fossil anatomy, trackways, in vivo locomotor data from extant taxa, kinematic simulation, dynamic simulation, and robotics. In mammals, Richards et al. (2021) used comparative ROM mapping and helical axis analysis to infer the unusual forelimb posture and function of the giant extinct marsupial *Palorchestes azael*, highlighting both the utility of quantitative ROM approaches and the difficulty of relying on direct one‐to‐one analogues in taxa with distinctive morphologies. These studies provide a useful context for the present framework, in which layer‐resolved ex vivo ROM is treated as one anatomical line of evidence within a broader morphofunctional calibration process.

The present study does not attempt to capture all six degrees of freedom. Instead, it provides a practical and anatomically explicit framework for quantifying how successive tissue layers modify a widely comparable axis of motion, passive flexion–extension, across mammalian postural types. In this context, the layer‐by‐layer results are best interpreted as empirical bounds rather than universal ‘correction factors’, because both the direction and magnitude of change are joint‐ and analogue‐dependent.

Figure 7 summarises a conceptual inverse framework linking three complementary evidence streams: osteological form, passive ex vivo mobility, and active in vivo movement. First, osteological form provides the anatomical template available from fossil and extant morphology, including articular geometry, segment proportions, lever arms, robusticity indices, geometric morphometrics, and finite element analysis. Second, passive ex vivo mobility provides tissue‐informed evidence on how anatomical layers and soft‐tissue effects modify the passive mobility envelope, generating anatomically grounded bounds rather than deterministic transformations of osteology‐only ROM. Third, active in vivo movement provides behavioural evidence on how part of this potential envelope is selected and coordinated during locomotor or functional tasks, through kinematics, kinetics, electromyography, inertial measurement units, and XROMM‐based approaches. These evidence streams can be interpreted alongside cautiously defined biological descriptors, such as posture, body mass, and independently supported functional or ecological context, which provide contextual grouping variables rather than direct predictors of ROM or criteria for single‐analogue selection. In combination, this framework supports joint‐, tissue‐ and context‐informed priors that can guide sensitivity analyses and parameter bracketing in musculoskeletal reconstructions, consistent with best‐practice discussions of ex vivo mobility measurement and interpretation (Manafzadeh, 2020). Importantly, passive ex vivo envelopes are not interpreted as direct estimates of in vivo functional maxima; rather, in vivo joint use is expected to occupy only part of the available passive envelope and to vary with task, posture and behavioural context (Granatosky et al., 2019; Medina‐González, 2026). This emphasis on tissue‐informed bounds, reciprocal calibration and explicit sensitivity testing is consistent with broader surveys showing that soft‐tissue reconstruction error is often non‐uniform and that sensitivity analysis remains inconsistently applied across palaeobiological biomechanical studies (Broyde et al., 2021).

Critically, the proposed integration does not imply that ex vivo passive maxima equal in vivo functional maxima. Rather, passive envelopes are treated as anatomically grounded limits, while stance‐phase utilisation provides a behavioural reference for how much of that envelope is ordinarily expressed under a standardised context. Nor does the framework imply that a single extant taxon can be treated as a direct analogue for a fossil species. Instead, it promotes an explicit comparison among morphological similarity, tissue‐informed passive bounds and observed in vivo angular use. By making each inferential step explicit and reversible in its assumptions, the workflow encourages transparent calibration of morphofunctional spaces while retaining appropriate caution for distal, multi‐element complexes. In such joints, osteology‐only states may be mechanically altered by changes in internal joint distance and alignment, which can shift the apparent mobility envelope even when osteological form is unchanged (Wiseman et al., 2022).

### 4.5 Limitations and future directions

The main limitations are (i) restricted taxon‐level replication, with one specimen per species, and (ii) a deliberate focus on flexion–extension. Consequently, the results are best interpreted as descriptive, hypothesis‐generating constraints on joint‐ and layer‐specific behaviour rather than as population‐level estimates. Increasing replication within taxa, and expanding coverage across postural and size extremes, will be necessary to test which aspects of the observed non‐monotonic trajectories are robust and which reflect individual variation, preservation state or joint‐specific handling demands.

A further constraint is that ex vivo passive end range is not equivalent to in vivo joint use, because active control, muscle tone, and task‐dependent loading can reshape the realised movement envelope. In vivo, proprioceptive feedback and neural control modulate muscle tone and task‐dependent co‐contraction, altering apparent joint stiffness and stability and thereby reshaping functional excursions relative to passive limits (Latash, 2018; Proske & Gandevia, 2012). Periarticular soft tissues also contribute not only passive restraint and guidance but elastic energy storage and joint stabilisation under load, and their functional behaviour depends on material properties that vary across ligaments and tendons (Alexander, 2002; Burgio et al., 2023; Summers & Koob, 2002). Importantly, the present ex vivo design does not attempt to substitute for in vivo kinematics. Instead, it isolates the mechanical contribution of successive tissue layers as an empirical component that is often treated as uniform in computational reconstructions. This is particularly relevant because modelling workflows commonly rely on generalised assumptions about soft tissues and joint properties across taxa and joints, both within musculoskeletal simulation platforms and within broader reconstruction pipelines, despite clear reasons to expect heterogeneity in periarticular organisation and mechanical behaviour among mammals (Damsgaard et al., 2006; Delp et al., 2007).

Methodologically, the restriction to a flexion–extension‐dominant movement trajectory in some joints and tissue conditions should be considered alongside the observed CV patterns, which indicate that endpoint detection can become ambiguous when multiple layers remain intact and when joint complexes permit coupled motions. This protocol therefore does not quantify the full six degrees of freedom of joint mobility, including coupled rotations, translations and long‐axis rotation, which are now recognised as important for reconstructing articular function (Kambic et al., 2014, 2017; Manafzadeh & Gatesy, 2021). In particular, unrestricted dry‐bone manipulation could be used in future sensitivity analyses to estimate how apparent flexion–extension changes when the operational movement plane, allowable translations, long‐axis rotation, abduction–adduction, and internal joint distance are no longer constrained by the congruence‐guided criteria applied in the present protocol.

Future work should therefore formalise evaluator training and reporting using established principles of arthrokinematics and osteokinematics from manual therapy and rehabilitation biomechanics, including standardised stabilisation, joint‐play concepts (accessory arthrokinematic translations and gliding motions), and explicit operational definitions of end feel, to reduce evaluator‐dependent variability while preserving anatomical realism (Kaltenborn, 2004; Neumann, 2017). Where appropriate, controlled loading and rate standardisation could be added to help separate evaluator‐dependent endpoint detection from genuine anatomical ambiguity.

Looking forward, Figure 7 can be developed into a scalable comparative framework by (i) adding more taxa to map how layer effects distribute across biological factors (posture, body mass, and functional or ecological context, when independently supported), and (ii) integrating these ex vivo constraints with in vivo kinematic datasets spanning behavioural faculties beyond walking, including running, jumping, climbing, and digging. This expansion would allow the morphofunctional logic to be applied across contexts, using comparative models based on multivariate morphological similarity, biological descriptors, and independent functional evidence, then contextualising osteology‐only estimates within tissue‐informed passive bounds that can be compared against task‐specific joint utilisation profiles.

Finally, extending the framework to incorporate multi‐axis motion and translations would align more closely with emerging expectations that palaeobiological reconstructions of articular function should explicitly address all six degrees of freedom (Manafzadeh & Gatesy, 2021), while preserving the practical value of layer‐resolved dissection experiments as an anatomically grounded constraint on mobility inference.

## 5 CONCLUSIONS

This study provides a layer‐resolved ex vivo characterisation of passive flexion–extension‐dominant ROM across six limb joints in four extant mammals spanning differing postural categories. Passive ROM did not expand uniformly with progressive tissue removal. Instead, responses were strongly joint‐ and taxon‐specific, with frequent intermediate‐condition maxima and, in several distal joints, reduced ROM following removal of the capsulo‐ligamentous envelope. These patterns show that osteology‐only measurements do not consistently represent an upper bound on biologically feasible passive flexion–extension‐dominant joint motion, because periarticular soft tissues can both constrain excursion and stabilise alignment within multi‐element joint complexes.

Between‐evaluator dispersion varied across joints and tissue conditions, tending to be higher in intact conditions and in joints that were more complex to manipulate consistently, particularly distal multi‐element joints. This variability is therefore informative for identifying where passive endpoints are intrinsically more ambiguous and where greater caution is warranted when interpreting absolute ROM values. Thus, measurement dispersion should be interpreted not only as methodological variation but also as a potential anatomical signal of endpoint ambiguity across tissue conditions.

Overall, the results support the use of layer‐resolved passive ROM as anatomically grounded, joint‐, tissue‐ and posture‐informed bounds for sensitivity analyses and morphofunctional interpretation. When integrated with comparative anatomical data, in vivo kinematic datasets and, where appropriate, computational models, this framework can help define plausible mobility envelopes without treating ex vivo passive maxima as direct predictors of in vivo joint use. In this way, layer‐resolved ex vivo ROM provides an anatomical line of evidence for refining morphofunctional interpretations of living taxa and supporting cautious palaeobiological applications.

## AUTHOR CONTRIBUTIONS

Conceptualisation: P.M.‐G.; Methodology: P.M.‐G., V.B.‐F., R.A.‐M., P.A.‐V.; Validation: P.M.‐G., P.A.‐V.; Investigation: P.M.‐G.; Resources: P.M.‐G., P.A.‐V, M.G.‐J.; Data curation: P.M.‐G., P.A.‐V; Formal analysis: P.M.‐G., V.B.‐F., R.A.‐M.; Software: P.M.‐G.; Writing – original draft: P.M.‐G.; Writing – review & editing: P.M.‐G., V.B.‐F., R.A.‐M., P.A.‐V., M.G.‐J.; Visualisation: P.M.‐G., V.B.‐F., R.A.‐M.; Project administration: P.M.‐G.; Funding acquisition: P.M.‐G.

## FUNDING INFORMATION

This research was funded by the National Agency for Research and Development (ANID), Chile, FONDECYT Iniciación en Investigación, grant number 11231111 (P.M.‐G.).

## CONFLICT OF INTEREST STATEMENT

The authors declare no conflicts of interest.

## ETHICS STATEMENT

All work was conducted on cadaveric specimens donated to the Laboratorio de Morfología Comparada (Comparative Morphology Laboratory), Universidad Austral de Chile for research and teaching purposes. No procedures were performed on live animals, and no animals were euthanised for this study. In accordance with institutional policy for research on animal cadavers, formal animal ethics committee review was not required. Access to and dissection of the donated specimens were authorised by the host laboratory under its institutional procedures. Specimen provenance, storage history, side dissected, and, when available, cause of death was recorded (Table 1; Supplementary File 1).

## Supporting information


**Supplementary File 1.** Layer‐resolved ex vivo joint ROM dataset and specimen metadata.
This dataset compiles the experimental and contextual information underpinning the study ‘Layer‐by‐layer soft‐tissue effects on flexion–extension‐dominant passive ex vivo limb joint ROM in quadrupedal mammals: an anatomical contribution to a morphofunctional framework’. It includes specimen metadata, handling conditions, anatomical descriptors, and layer‐resolved flexion–extension ROM measurements obtained from four extant mammals spanning differing postural categories. Each row corresponds to a unique combination of specimen, limb (forelimb/hindlimb), joint, anatomical axis and tissue‐layer condition: intact (S+M+CL+O), myofascial (M+CL+O), capsulo‐ligamentous (CL+O) and osteology‐only (O). By convention, negative values denote extension and positive values denote flexion. For each joint and tissue‐layer condition, the file provides summary statistics, including mean, standard deviation, and percentage change relative to the intact baseline condition. Contextual biological descriptors, such as body mass and posture category, are included to support comparative anatomical interpretation of interspecific differences in layer‐resolved ROM patterns. These descriptors are intended as broad contextual variables, not as formal predictors of ROM or as criteria for direct analogue selection. Note. Percentage change was calculated relative to the intact S+M+CL+O condition, which served as the baseline reference. Accordingly, values of 0 in the S+M+CL+O rows indicate the reference state. Positive values indicate increased ROM relative to this baseline, whereas negative values indicate reduced ROM.

**Supplementary File 2.** Repeatability pilot for maximal extension endpoints in a juvenile alpaca hindlimb (ex vivo ROM, by tissue‐layer condition). For each joint and tissue condition (S+M+CL+O, M+CL+O, CL+O, O), two evaluators (R1–R2) obtained three repeated measurements (M1–M3) of maximal extension range of motion (ROM, °). Within‐evaluator variability is reported as mean (X), standard deviation (SD) and intra‐rater coefficient of variation (CV, %). Between‐evaluator differences are summarised as inter‐rater CV (%) and absolute mean differences (Δ, °). Inter‐rater agreement was assessed using a two‐way mixed‐effects model with absolute agreement, reporting ICC (3,1) with 95% confidence intervals (single measures); ICC (3,2) is also provided for the average of two evaluators. Note. This file reports the reliability assessment used to evaluate consistency during passive manipulation and digital angle extraction. Reliability metrics are presented as methodological quality control indicators for the layer‐resolved ROM protocol. The main dataset was obtained from three trained evaluators per joint and tissue condition.

**Supplementary File 3.** Representative image set for layer‐resolved passive flexion–extension‐dominant ROM measurements.

Video S1.

## Data Availability

All data supporting the findings of this study are provided in the main text and associated supplementary files. The complete dataset and supplementary materials are openly available in Zenodo (DOI: https://doi.org/10.5281/zenodo.18671050). The repository includes the full layer‐resolved ex vivo joint range‐of‐motion dataset (flexion–extension), specimen metadata and anatomical descriptors, joint‐ and condition‐level summary statistics (mean, SD and percentage change across tissue‐layer conditions), repeatability outputs from the juvenile alpaca pilot (within‐ and between‐evaluator variability and ICC estimates), representative image documentation of end‐range positions used for digitisation, and Supplementary Video 1 documenting representative handling and joint manipulation (chilla fox shoulder under the S+M+CL condition).
