# Supplementary material for: Layer‐by‐layer soft‐tissue effects on flexion–extension‐dominant passive ex vivo limb joint ROM in quadrupedal mammals: An anatomical contribution to a morphofunctional framework
Source: J Anat. 2026 Jun 30:10.1111/joa.70207. Online ahead of print. doi: 10.1111/joa.70207 (PMC13398786; doi:10.1111/joa.70207)
Supplement: Supplementary file 3 — Representative image set for layer‐resolved passive flexion–extension‐dominant ROM measurements. [file JOA-9999-0-s004.pdf]

Layer-by-layer soft-tissue effects on flexion–extension-dominant passive ex vivo limb joint ROM in quadrupedal mammals: an anatomical contribution to a morphofunctional framework

Supplementary File 3. Representative image set for layer-resolved passive flexion–extension-dominant ROM measurements. This file provides representative still images documenting the flexion and extension endpoints used to quantify passive ROM for each joint and tissue condition. For each species and joint, one representative flexion endpoint and one representative extension endpoint are shown across the full dissection sequence: S+M+CL+O, M+CL+O, CL+O and O. Images include the calibrated digital angle overlays and proximal and distal segment reference lines used for angle digitisation in Tracker v6.1.6. The general landmarking logic, including functional centroids, segmental vectors and the body-referenced coordinate system for proximal joints, is explained in Fig. 1 and in the Methods. The primary outcome was total ROM, calculated as the angular span between flexion and extension endpoints, rather than absolute joint pose. Black boxes were added for ethical presentation and do not obscure the anatomical regions or reference lines used for digitisation. Abbreviations: S, skin and subcutaneous tissue; M, myofascia; CL, capsulo-ligamentous tissues; O, osteology only; ROM, range of motion.

Shoulder Flexion and Extension of Rabbit (*Oryctolagus cuniculus*)

**Flexion**			
**S + M+ CL + O**	**M+ CL + O**	**CL + O**	**O**
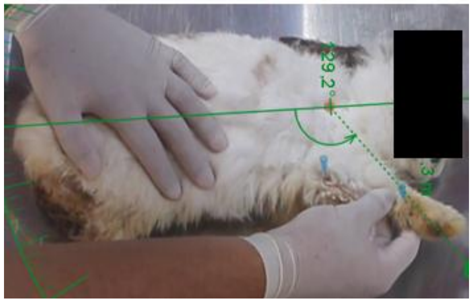	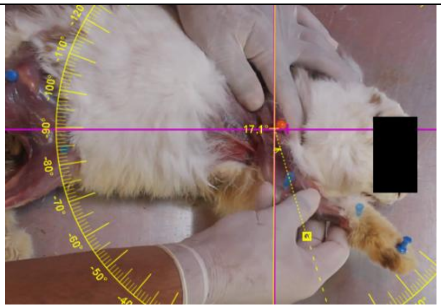	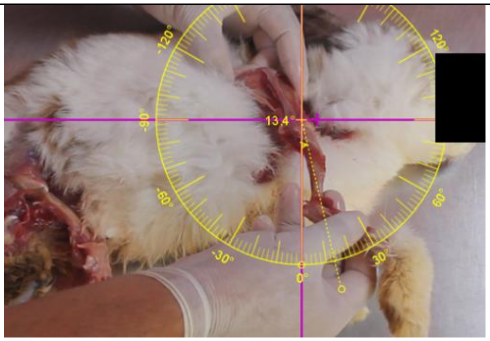	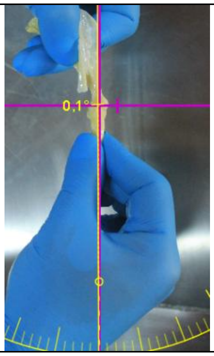
**Extension**			
**S + M+ CL + O**	**M+ CL + O**	**CL + O**	**O**
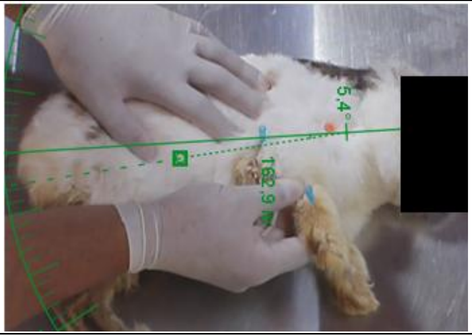	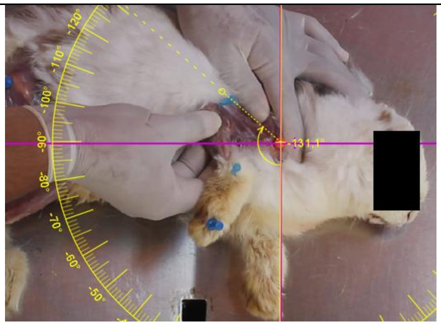	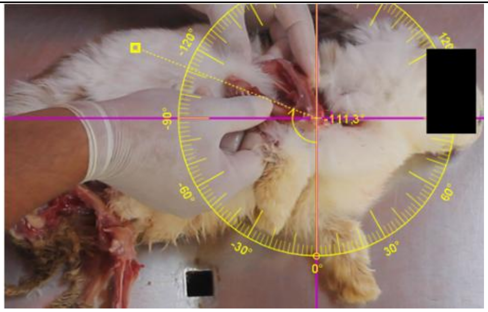	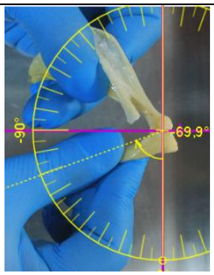

Elbow Flexion and Extension of Rabbit (*Oryctolagus cuniculus*)

**Flexion**			
**S + M+ CL + O**	**M+ CL + O**	**CL + O**	**O**
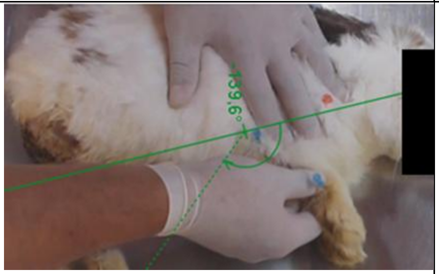	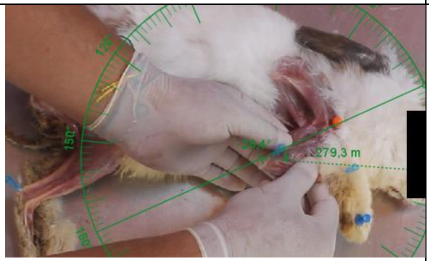	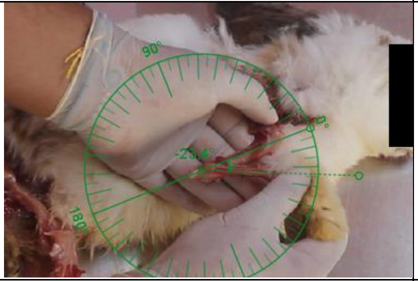	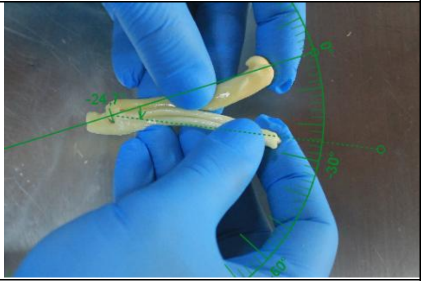
**Extension**			
**S + M+ CL + O**	**M+ CL + O**	**CL + O**	**O**
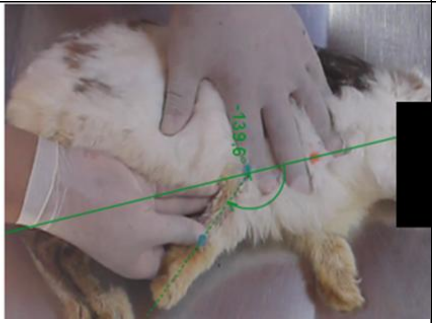	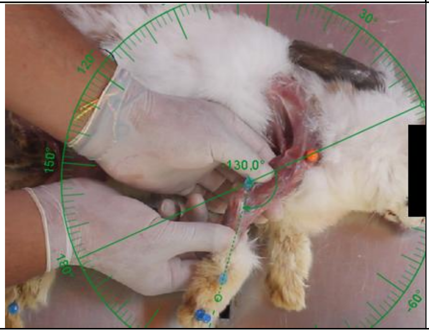	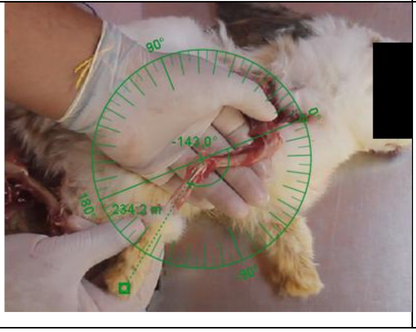	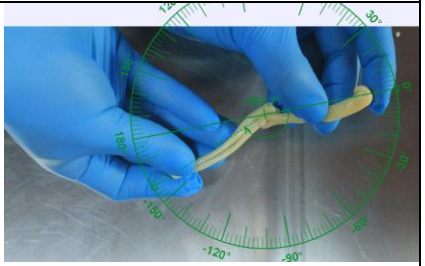

Wrist Flexion and Extension of Rabbit (*Oryctolagus cuniculus*)

Flexion

S + M+ CL + O

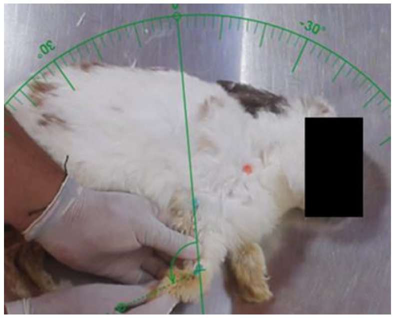

M+ CL + O

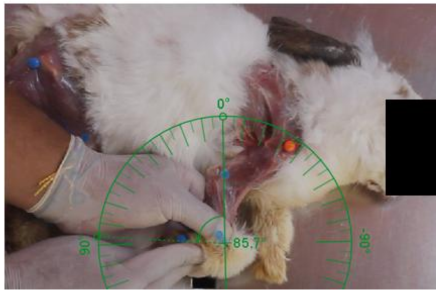

CL + O

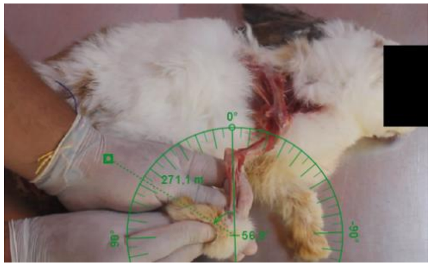

O

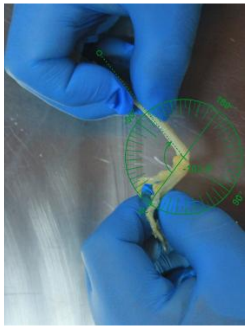

Extension

S + M+ CL + O

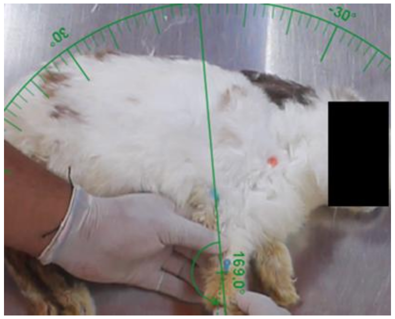

M+ CL + O

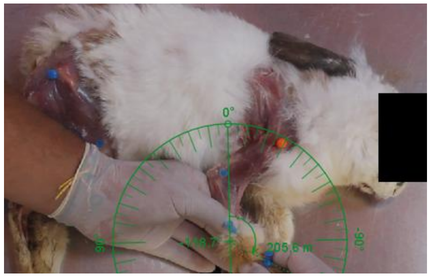

CL + O

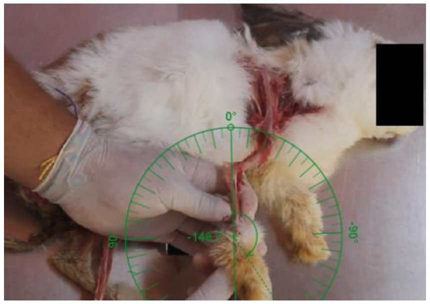

O

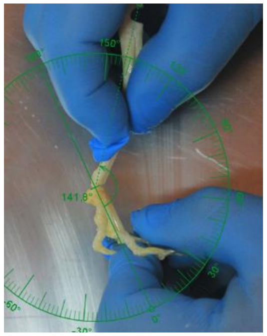

Hip Flexion and Extension of Rabbit (*Oryctolagus cuniculus*)

**Flexion**			
**S + M+ CL + O**	**M+ CL + O**	**CL + O**	**O**
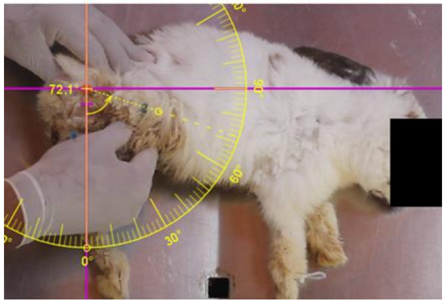	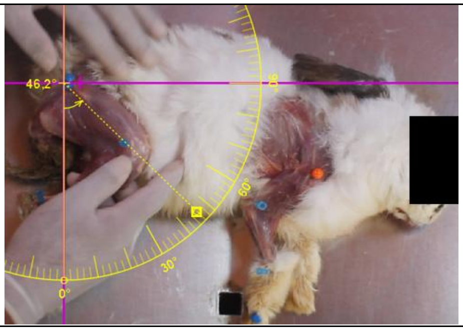	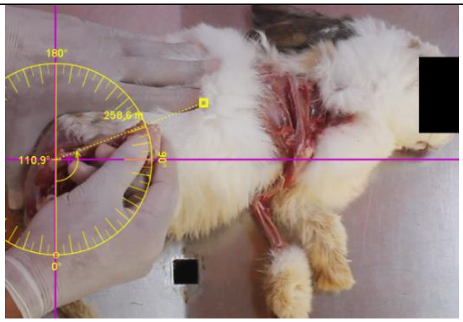	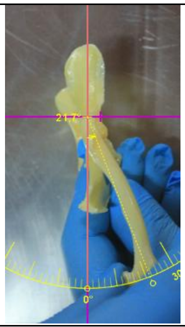
**Extension**			
**S + M+ CL + O**	**M+ CL + O**	**CL + O**	**O**
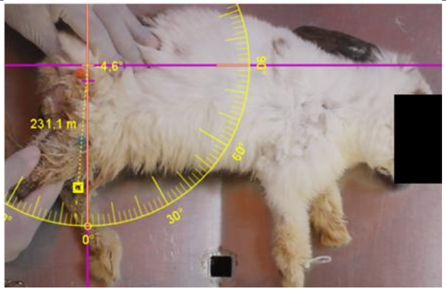	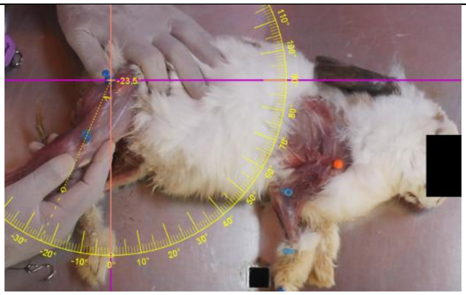	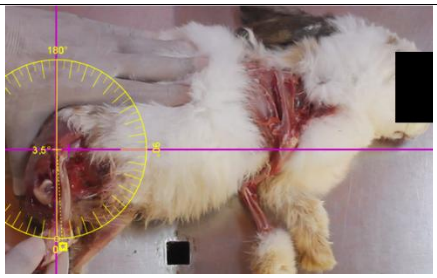	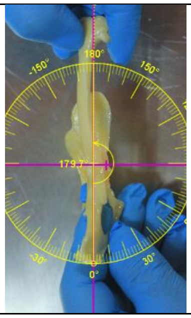

Knee Flexion and Extension of Rabbit (*Oryctolagus cuniculus*)

**Flexion**			
**S + M+ CL + O**	**M+ CL + O**	**CL + O**	**O**
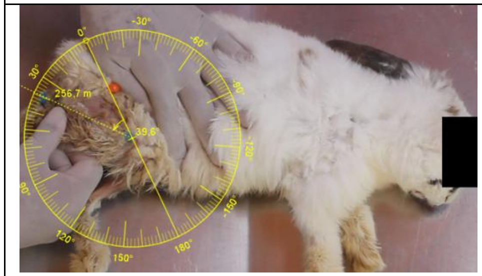	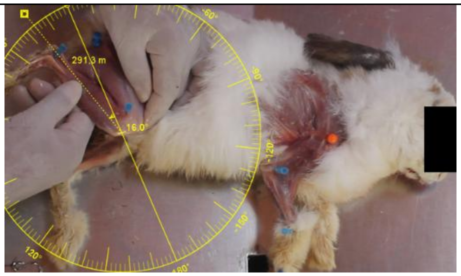	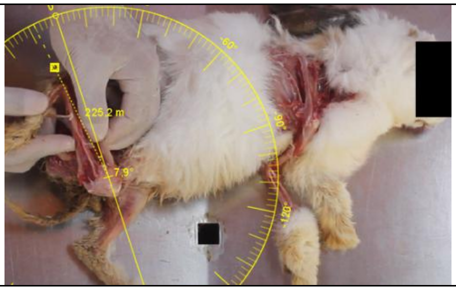	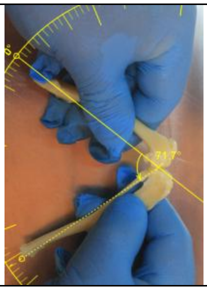
**Extension**			
**S + M+ CL + O**	**M+ CL + O**	**CL + O**	**O**
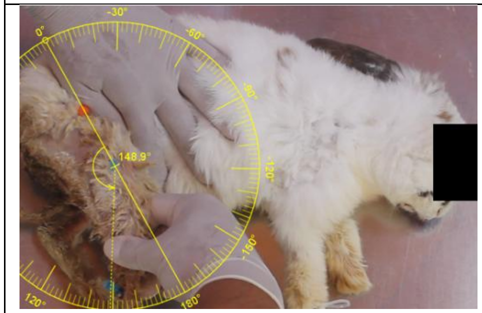	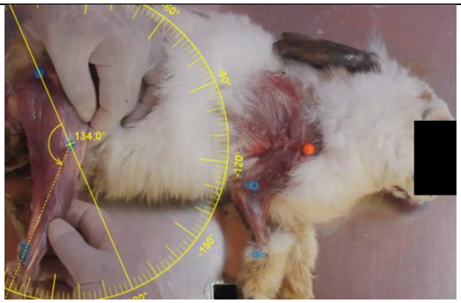	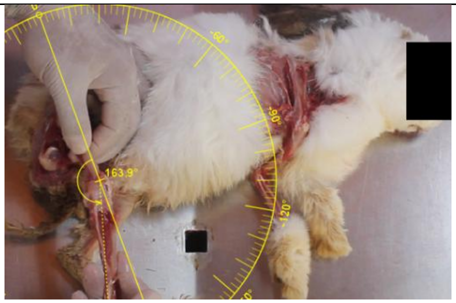	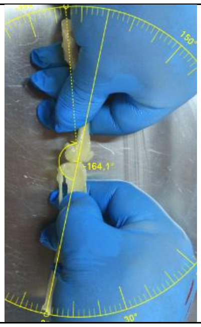

Ankle Flexion and Extension of Rabbit (*Oryctolagus cuniculus*)

**Flexion**			
**S + M+ CL + O**	**M+ CL + O**	**CL + O**	**O**
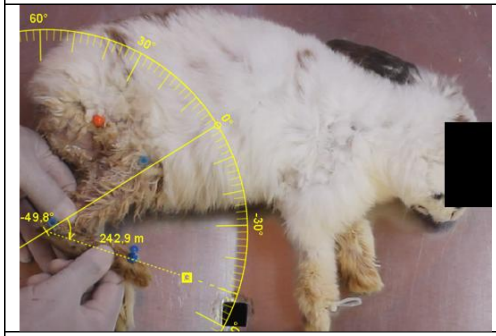	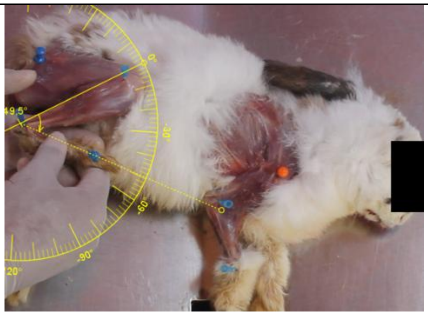	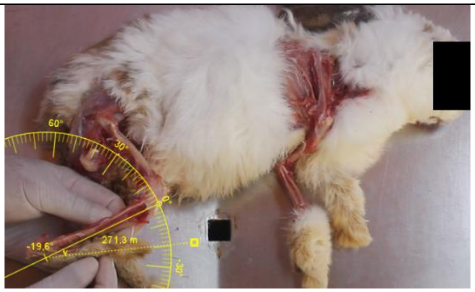	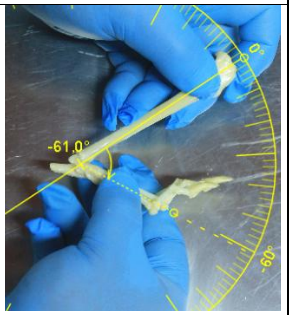
**Extension**			
**S + M+ CL + O**	**M+ CL + O**	**CL + O**	**O**
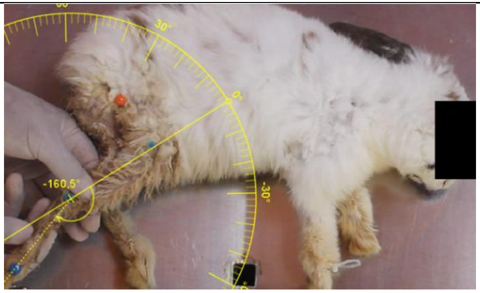	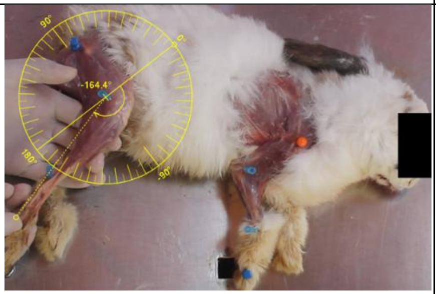	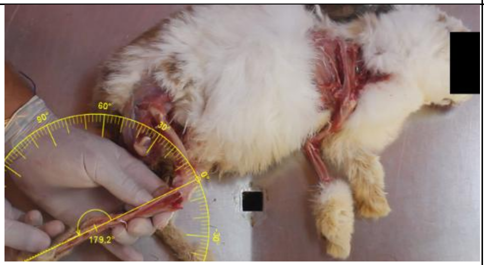	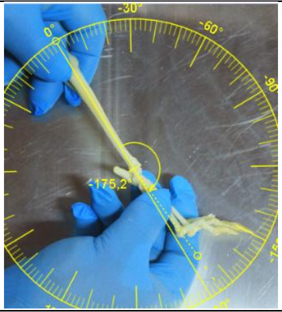

Shoulder Flexion and Extension of Pudu (*Pudu puda*)

**Flexion**			
**S + M+ CL + O**	**M+ CL + O**	**CL + O**	**O**
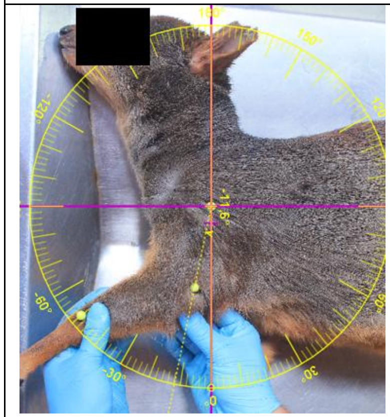	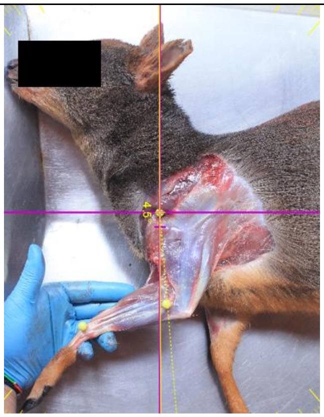	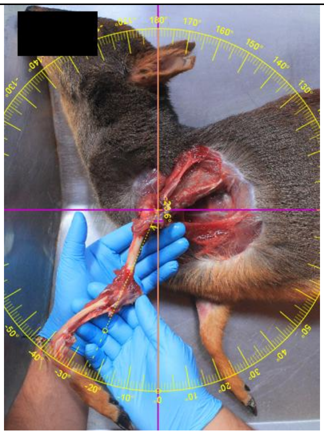	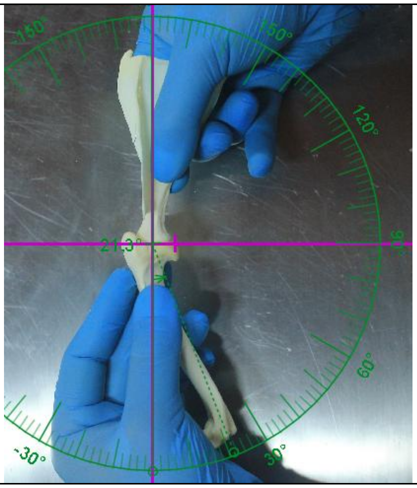
**Extension**			
**S + M+ CL + O**	**M+ CL + O**	**CL + O**	**O**
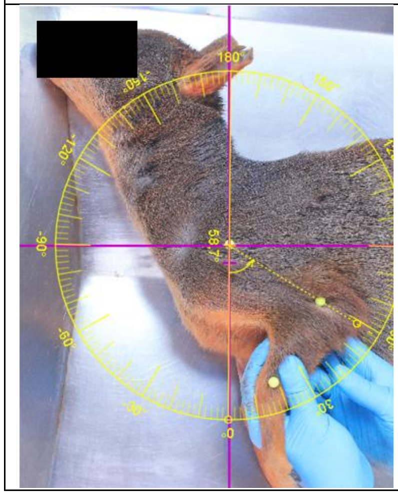	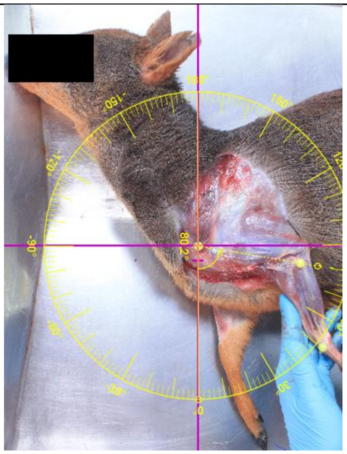	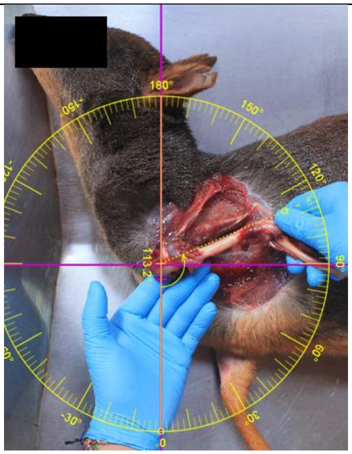	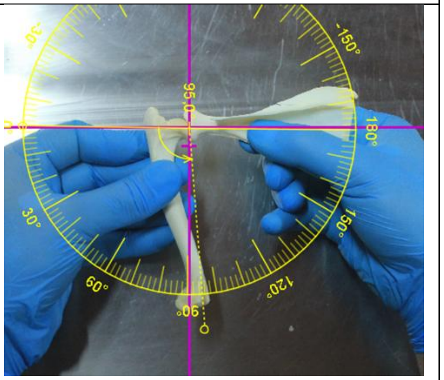

Elbow Flexion and Extension of Pudu (*Pudu puda*)

**Flexion**			
**S + M+ CL + O**	**M+ CL + O**	**CL + O**	**O**
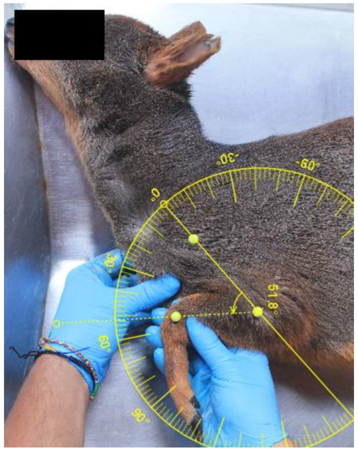	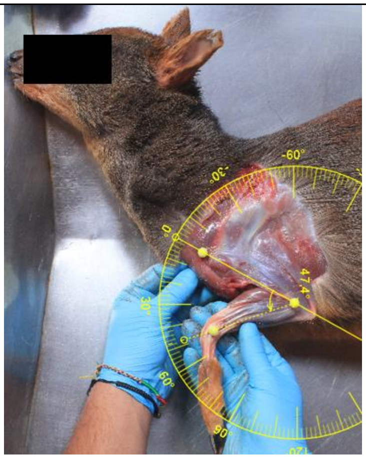	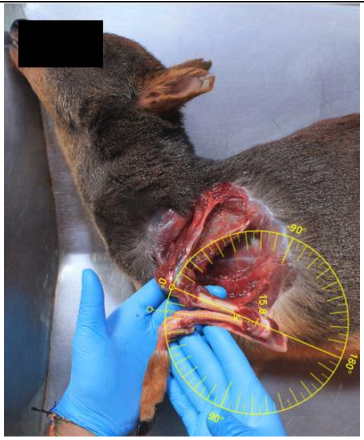	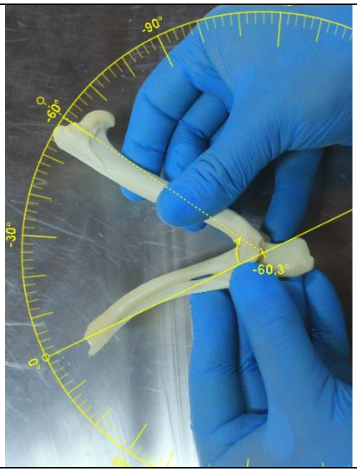
**Extension**			
**S + M+ CL + O**	**M+ CL + O**	**CL + O**	**O**
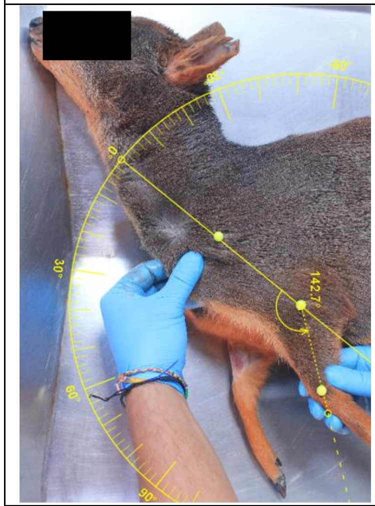	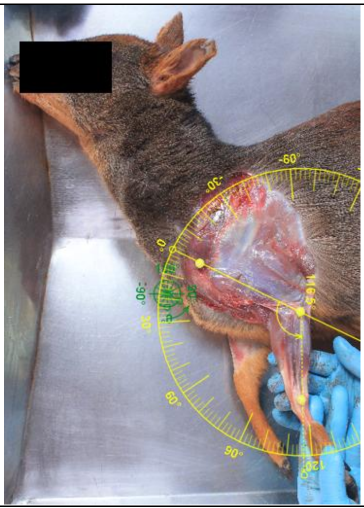	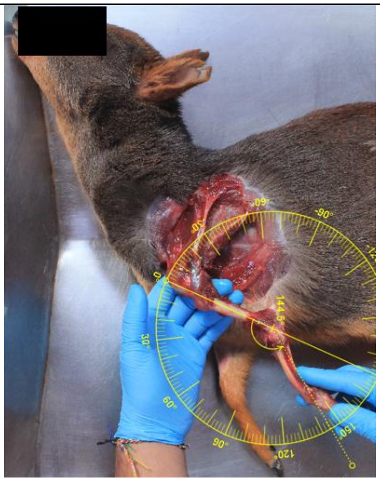	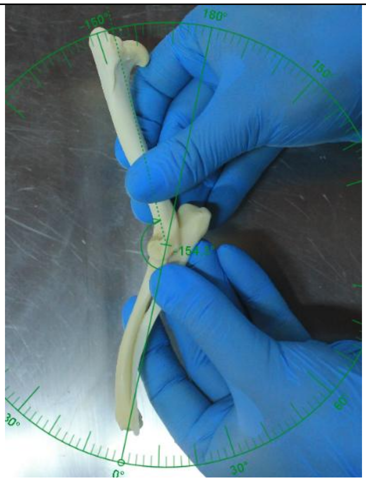

Wrist Flexion and Extension of Pudu (*Pudu puda*)

**Flexion**			
**S + M+ CL + O**	**M+ CL + O**	**CL + O**	**O**
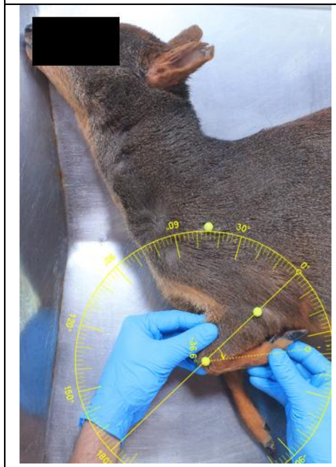	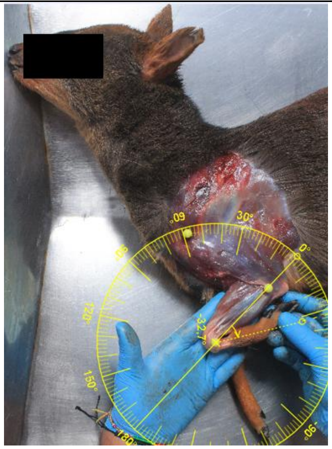	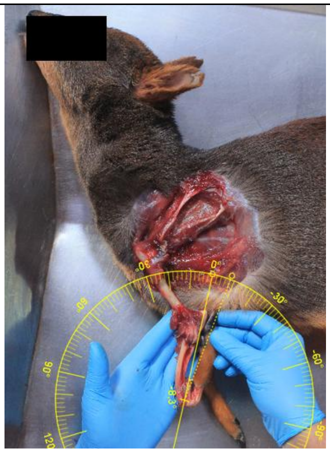	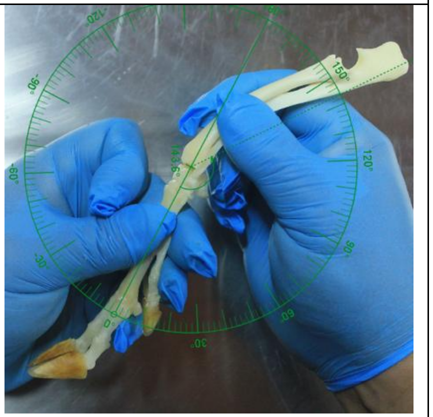
**Extension**			
**S + M+ CL + O**	**M+ CL + O**	**CL + O**	**O**
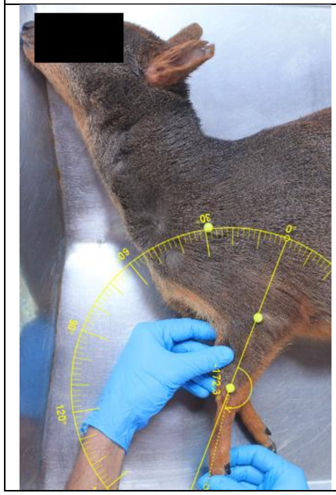	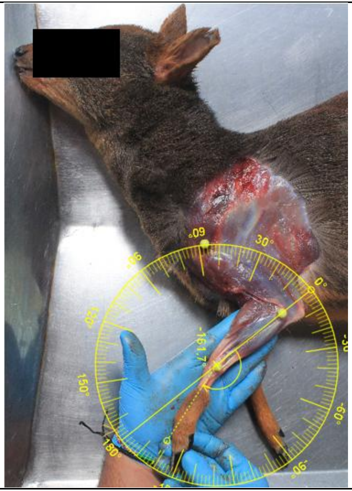	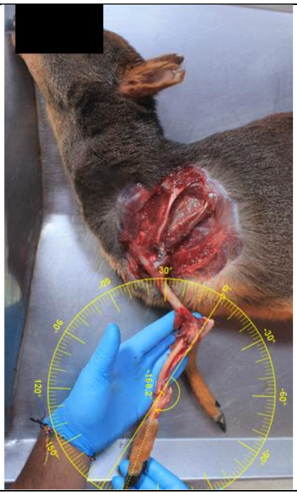	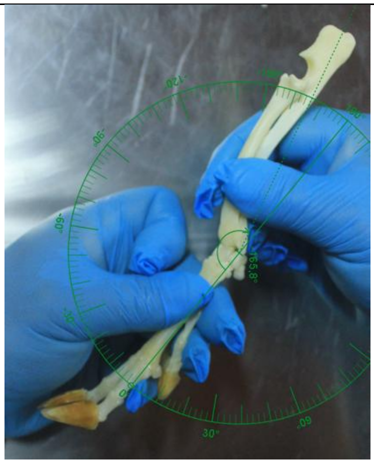

Hip Flexion and Extension of Pudu (*Pudu puda*)

Flexion			
**S + M+ CL + O**	**M+ CL + O**	**CL + O**	**O**
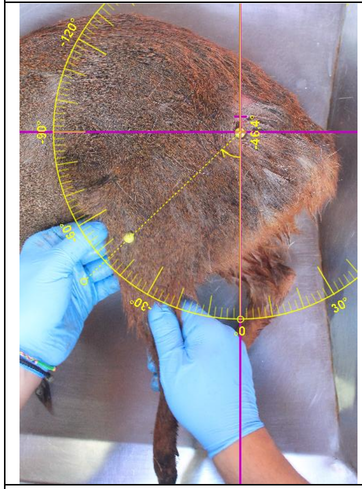	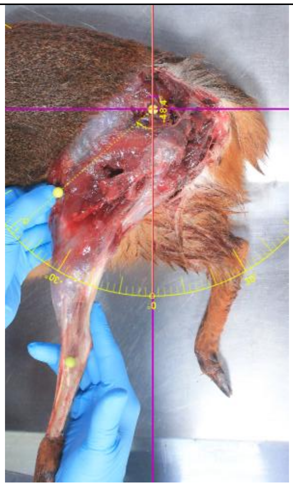	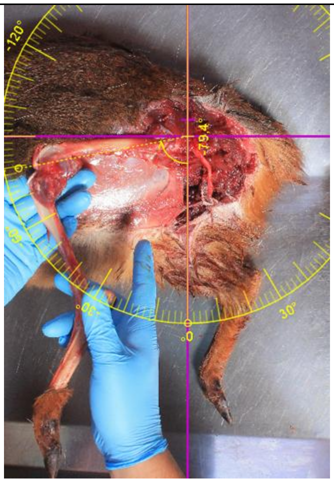	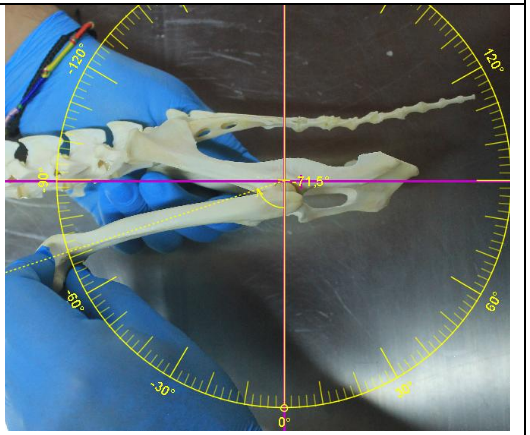
**Extension**			
**S + M+ CL + O**	**M+ CL + O**	**CL + O**	**O**
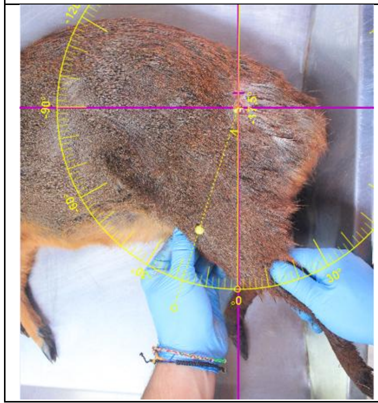	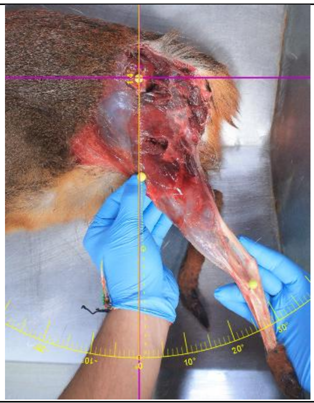	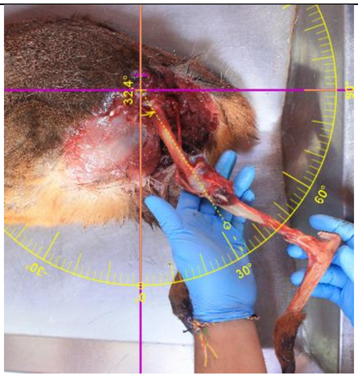	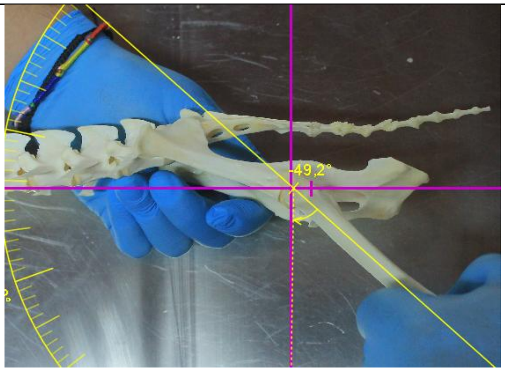

Knee Flexion and Extension of Pudu (*Pudu puda*)

**Flexion**			
**S + M+ CL + O**	**M+ CL + O**	**CL + O**	**O**
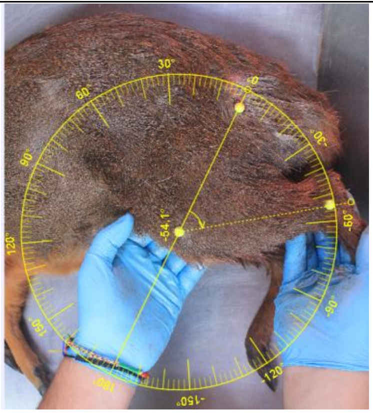	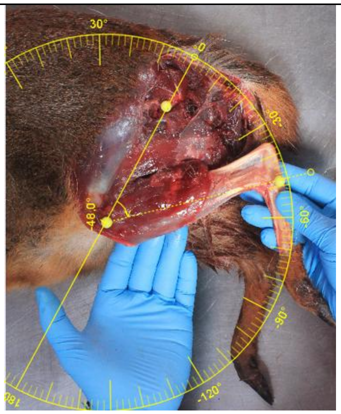	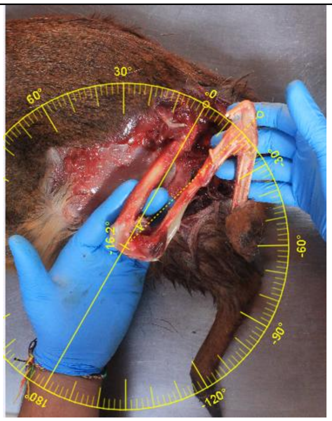	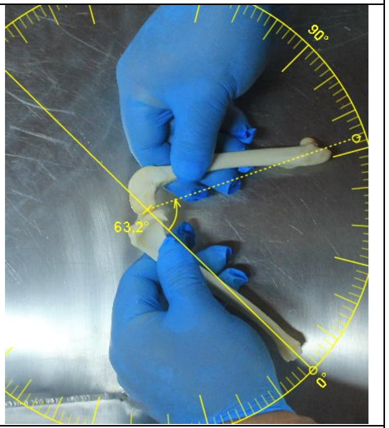
**Extension**			
**S + M+ CL + O**	**M+ CL + O**	**CL + O**	**O**
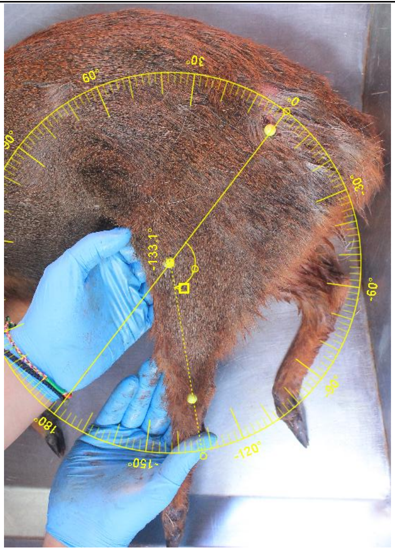	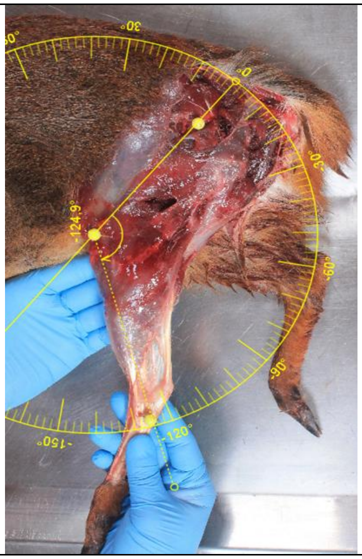	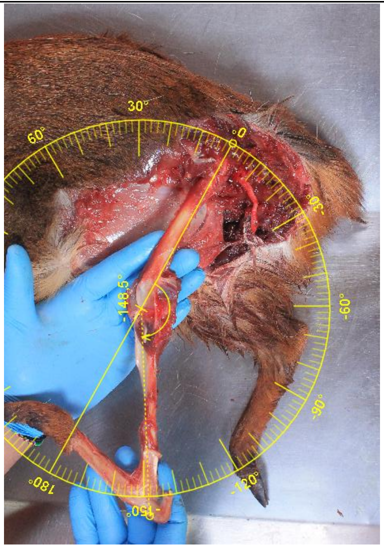	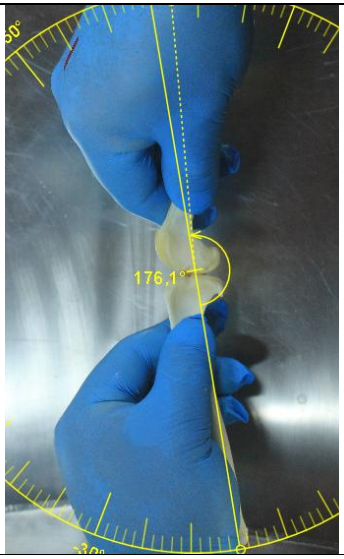

Ankle Flexion and Extension of Pudu (*Pudu puda*)

Flexion			
**S + M+ CL + O**	**M+ CL + O**	**CL + O**	**O**
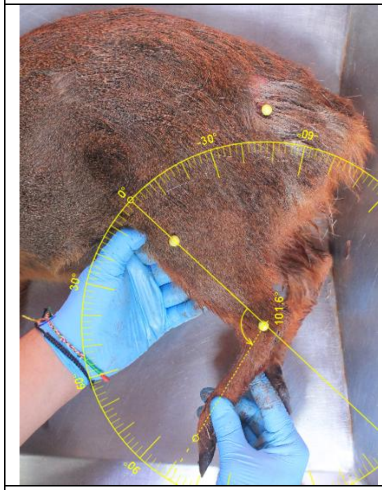	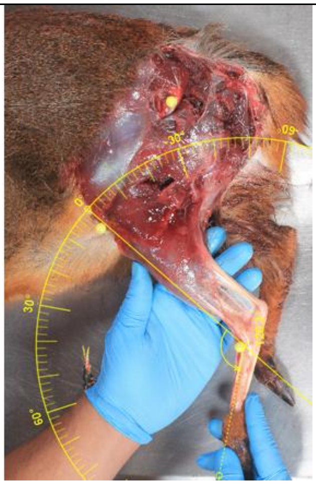	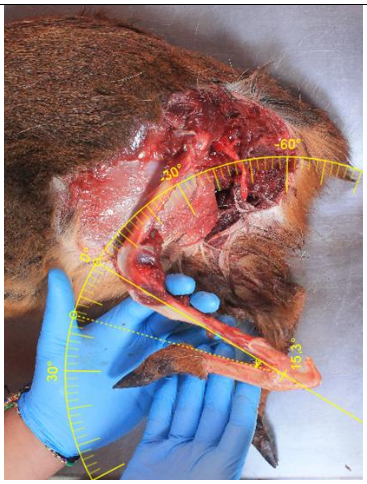	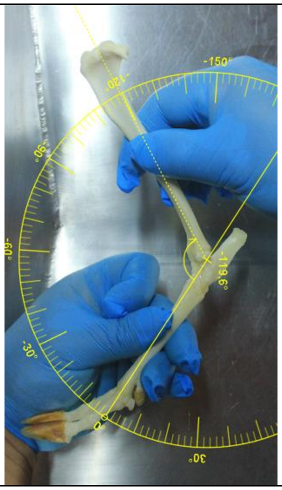
**Extension**			
**S + M+ CL + O**	**M+ CL + O**	**CL + O**	**O**
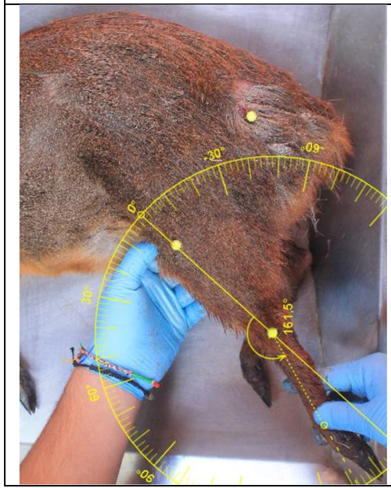	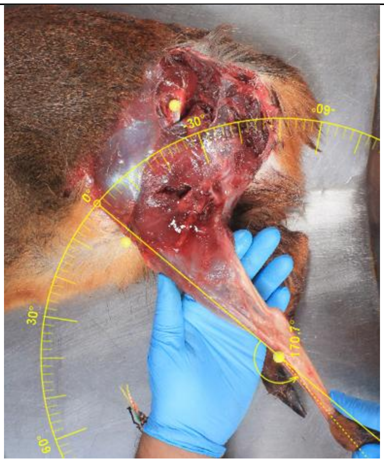	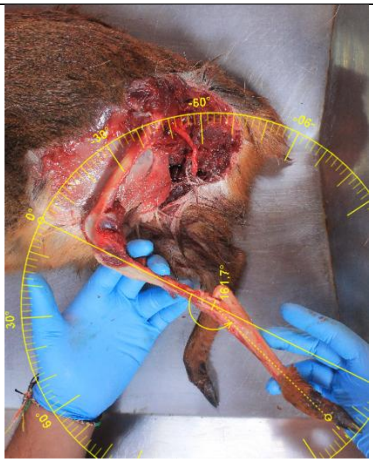	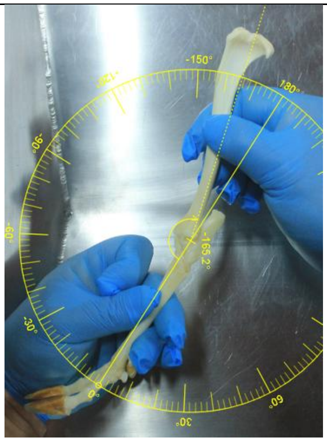

Shoulder Flexion and Extension of Chilla fox (*Lycalopex griseus*)

**Flexion**			
**S + M+ CL + O**	**M+ CL + O**	**CL + O**	**O**
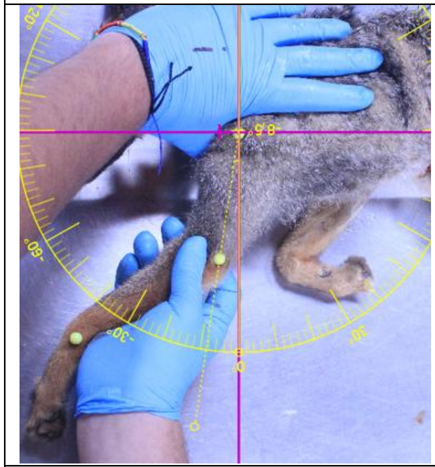	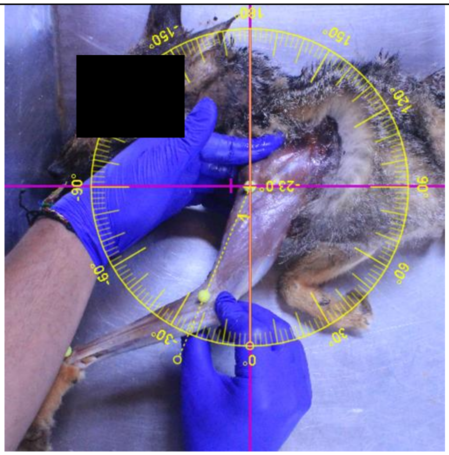	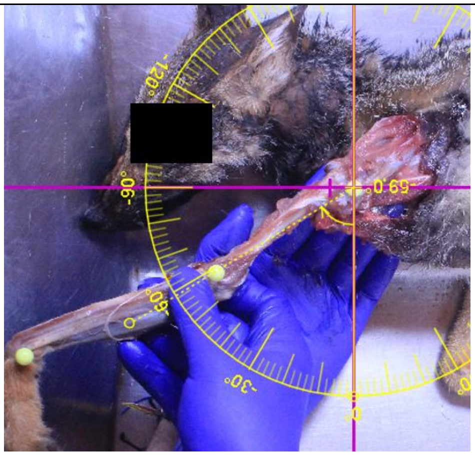	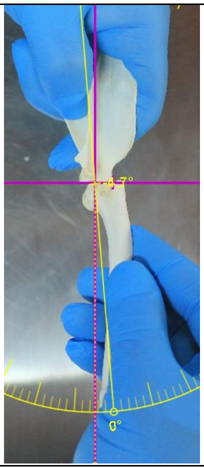
**Extension**			
**S + M+ CL + O**	**M+ CL + O**	**CL + O**	**O**
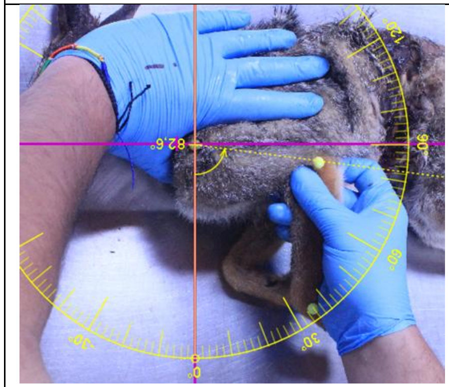	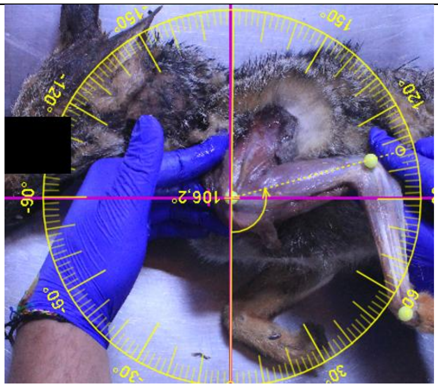	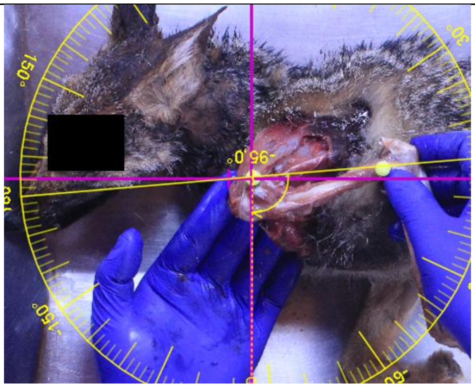	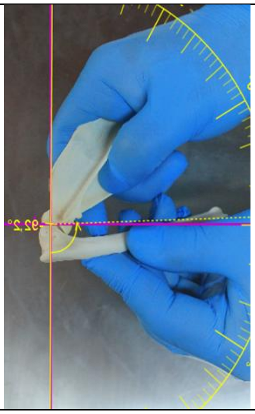

Elbow Flexion and Extension of Chilla fox (*Lycalopex griseus*)

Flexion			
S + M+ CL + O	M+ CL + O	CL + O	O
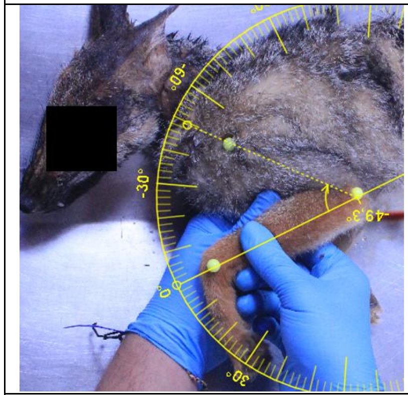	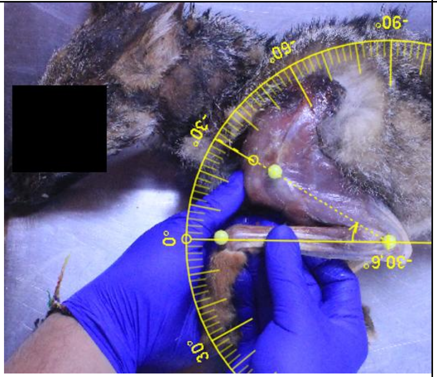	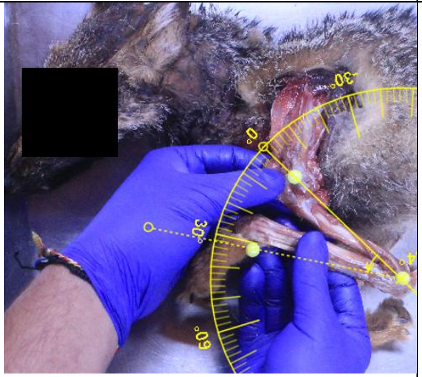	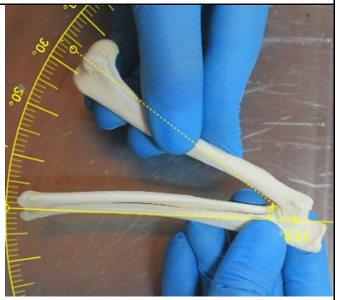
**Extension**			
S + M+ CL + O	M+ CL + O	CL + O	O
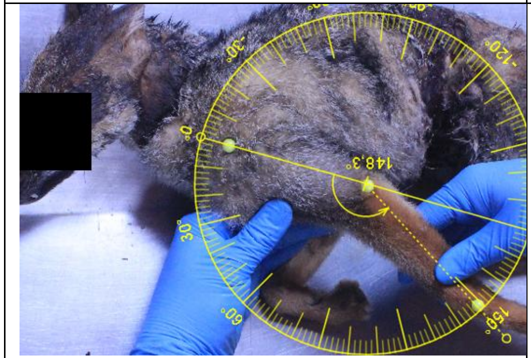	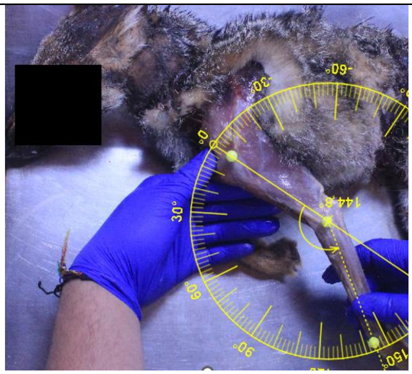	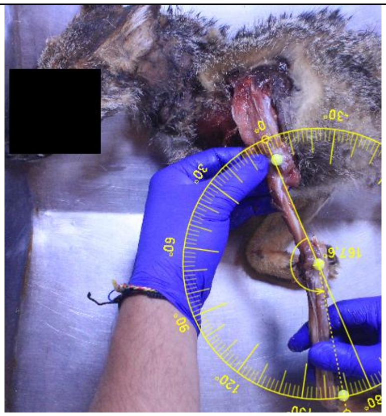	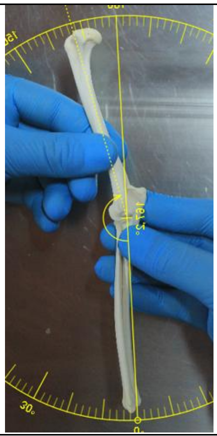

Wrist Flexion and Extension of Chilla fox (*Lycalopex griseus*)

**Flexion**			
**S + M+ CL + O**	**M+ CL + O**	**CL + O**	**O**
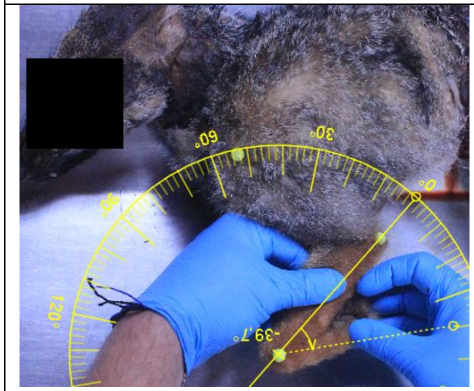	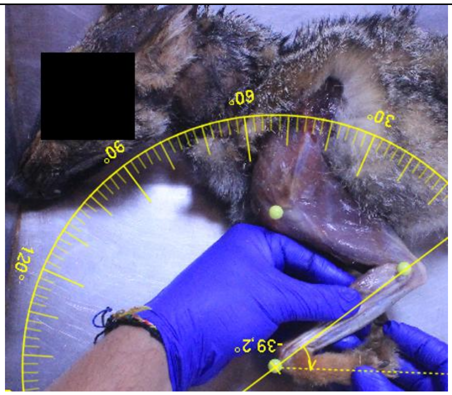	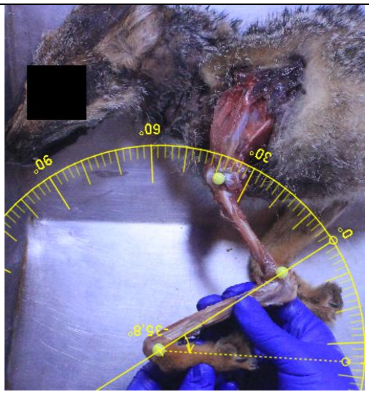	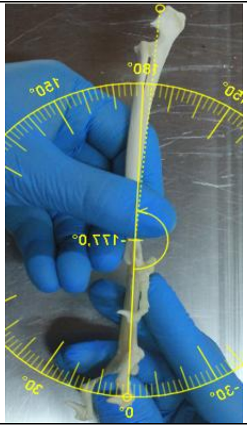
**Extension**			
**S + M+ CL + O**	**M+ CL + O**	**CL + O**	**O**
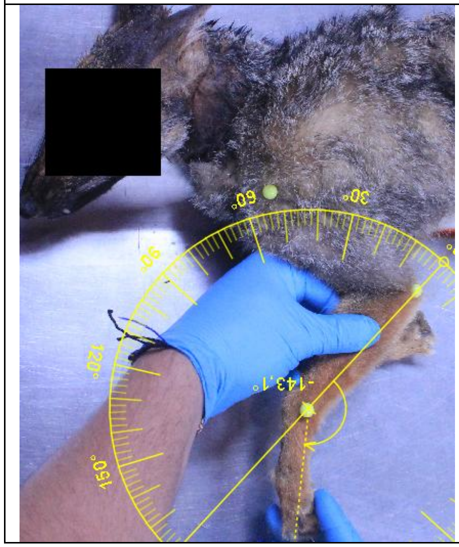	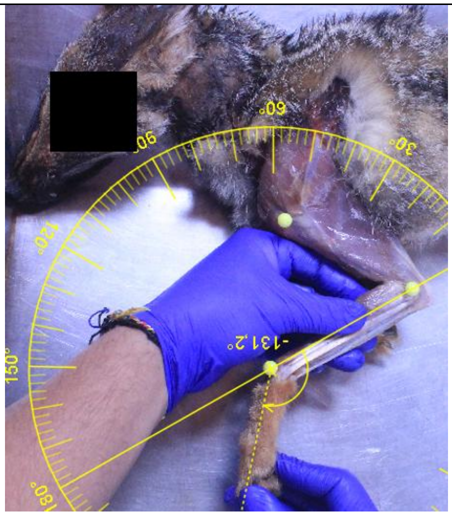	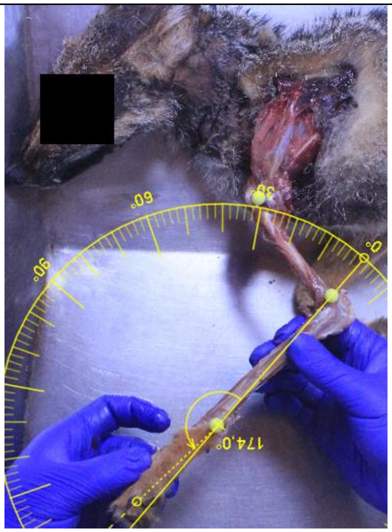	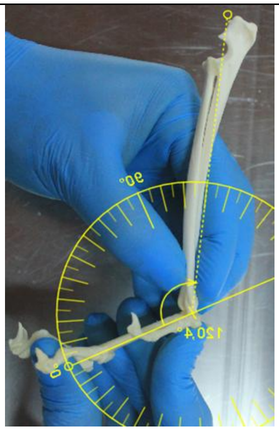

Hip Flexion and Extension of Chilla fox (*Lycalopex griseus*)

Flexion			
**S + M+ CL + O**	**M+ CL + O**	**CL + O**	**O**
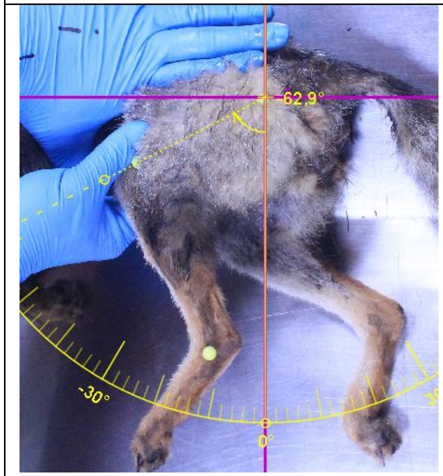	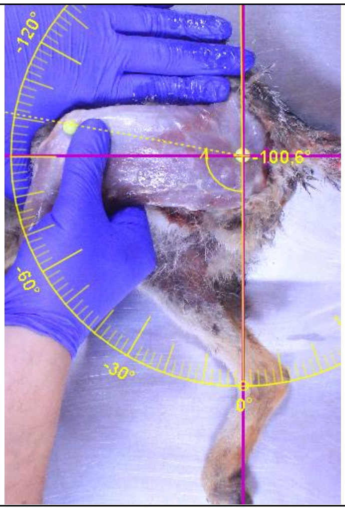	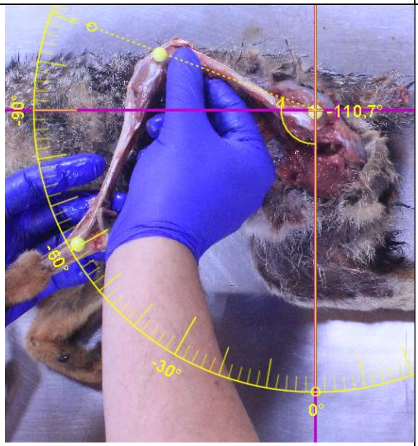	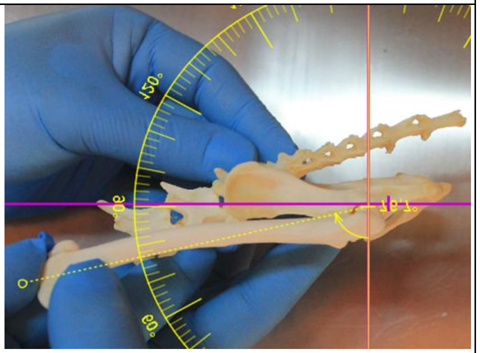
**Extension**			
**S + M+ CL + O**	**M+ CL + O**	**CL + O**	**O**
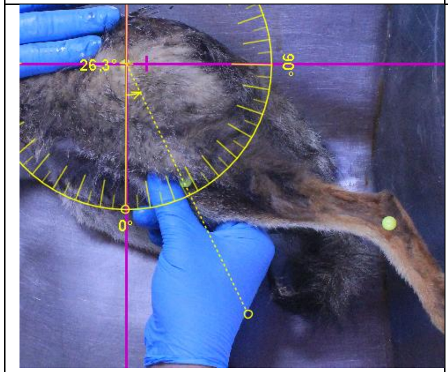	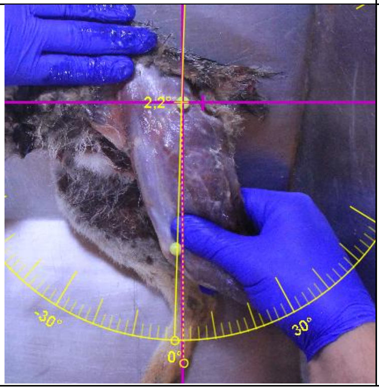	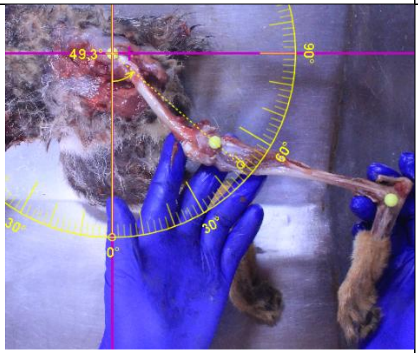	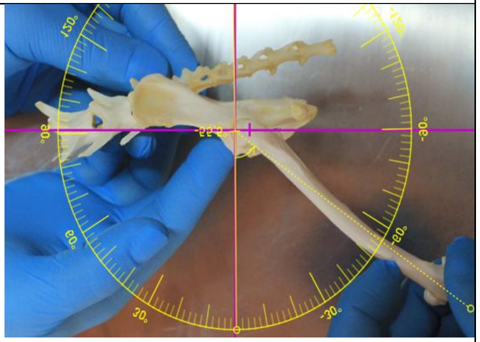

Knee Flexion and Extension of Chilla fox (*Lycalopex griseus*)

**Flexion**			
**S + M+ CL + O**	**M+ CL + O**	**CL + O**	**O**
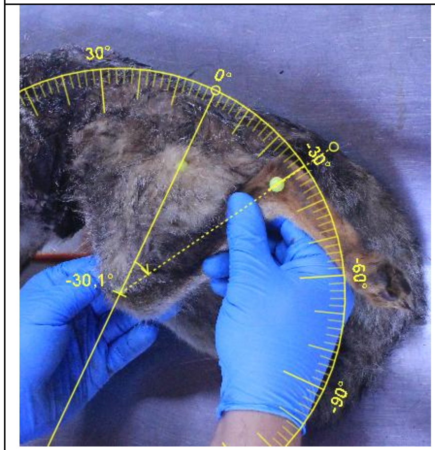	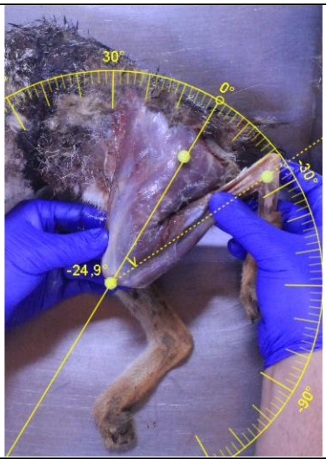	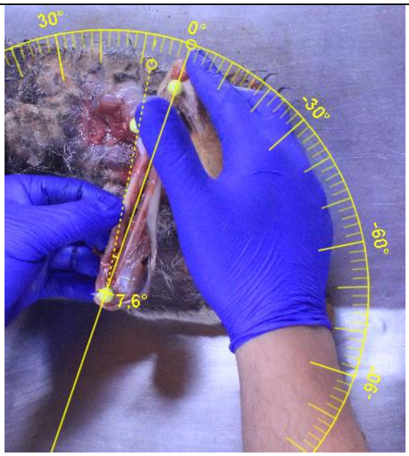	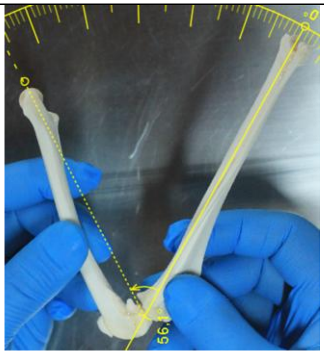
**Extension**			
**S + M+ CL + O**	**M+ CL + O**	**CL + O**	**O**
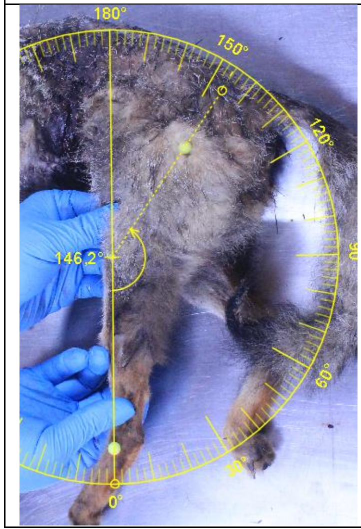	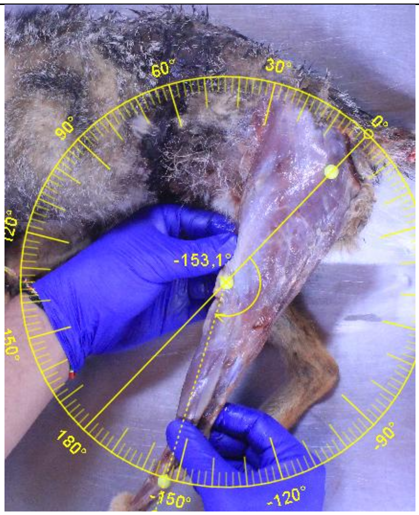	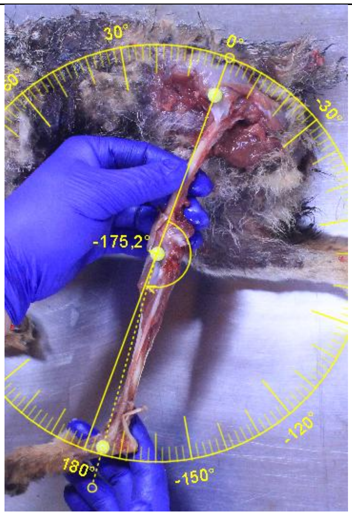	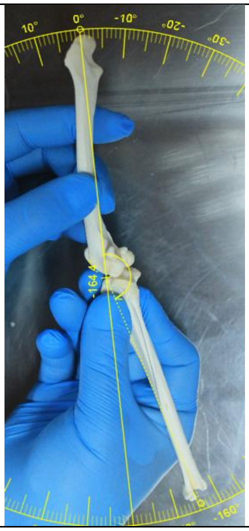

Ankle Flexion and Extension of Chilla fox (*Lycalopex griseus*)

Flexion			
S + M+ CL + O	M+ CL + O	CL + O	O
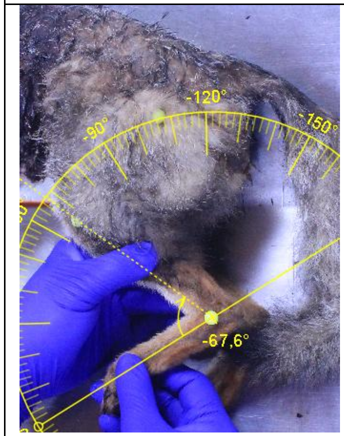	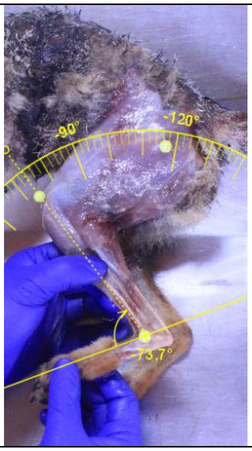	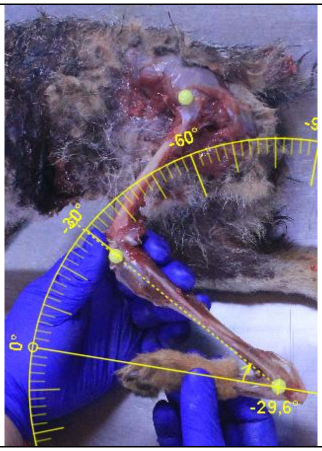	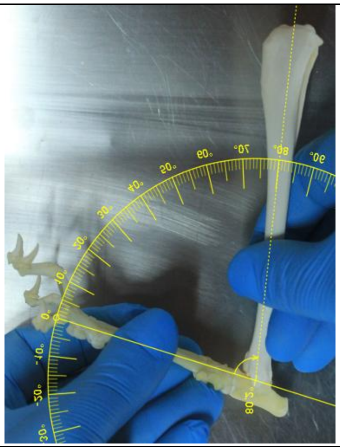
**Extension**			
S + M+ CL + O	M+ CL + O	CL + O	O
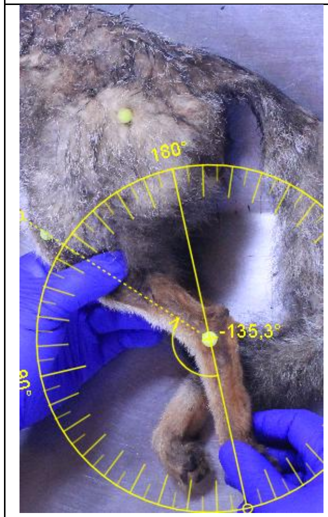	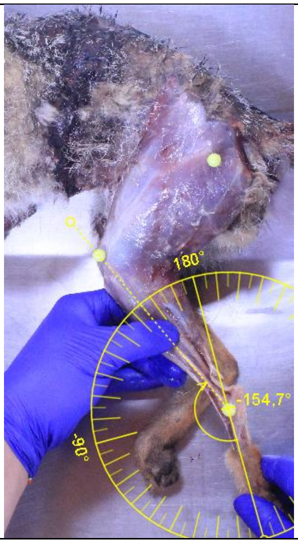	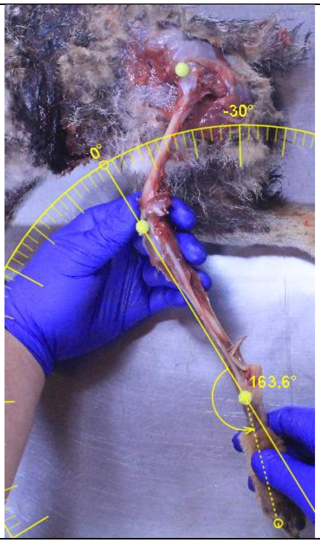	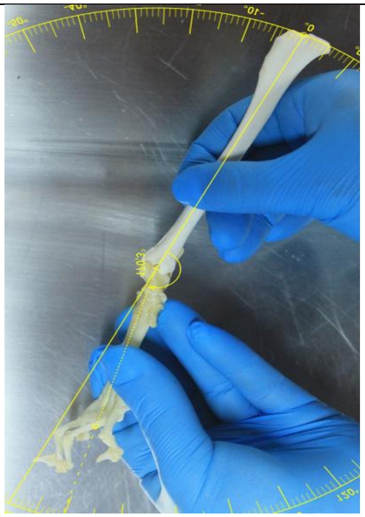

Shoulder Flexion and Extension of Pig (*Sus scrofa*)

**Flexion**			
**S + M+ CL + O**	**M+ CL + O**	**CL + O**	**O**
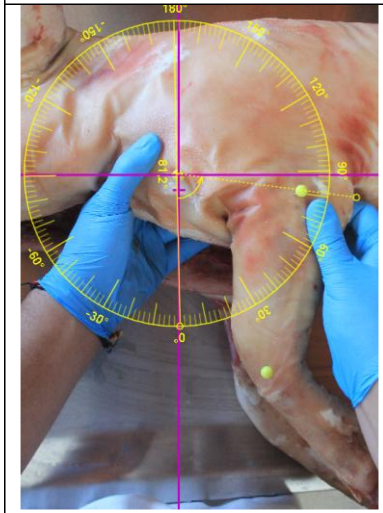	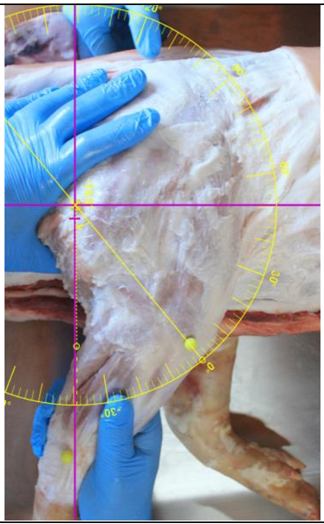	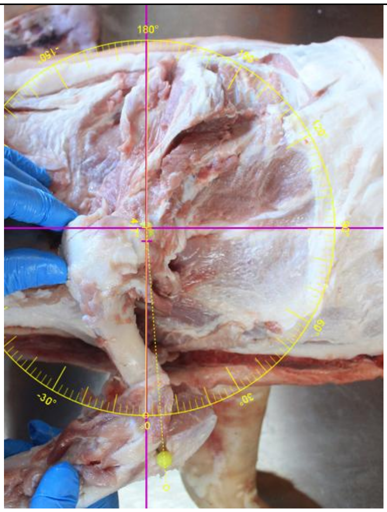	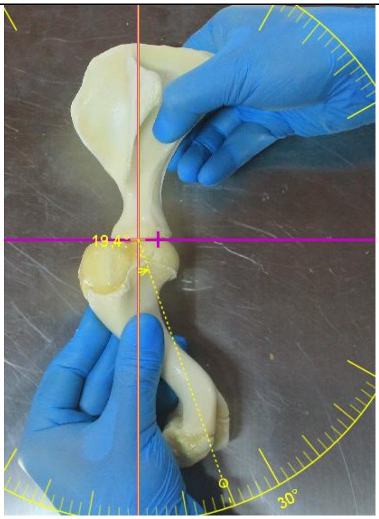
**Extension**			
**S + M+ CL + O**	**M+ CL + O**	**CL + O**	**O**
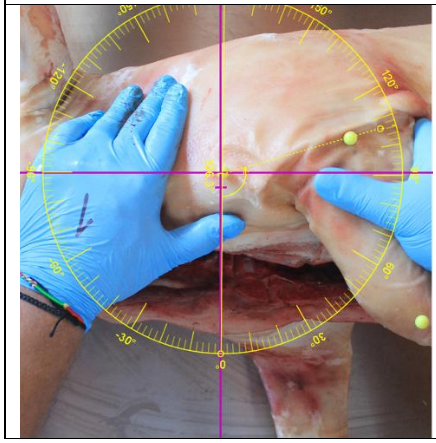	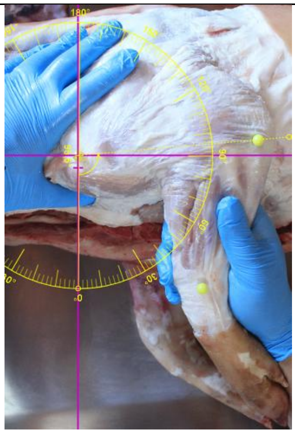	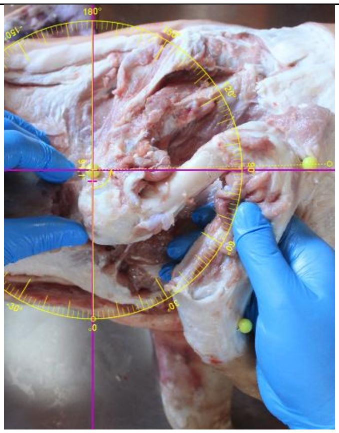	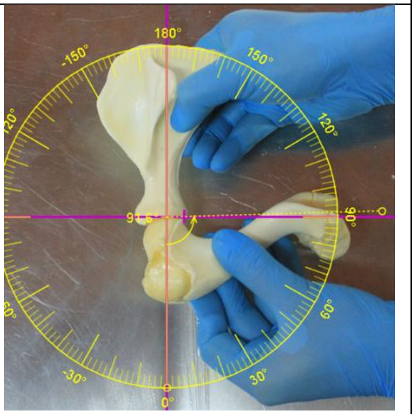

Elbow Flexion and Extension of Pig (*Sus scrofa*)

**Flexion**			
**S + M+ CL + O**	**M+ CL + O**	**CL + O**	**O**
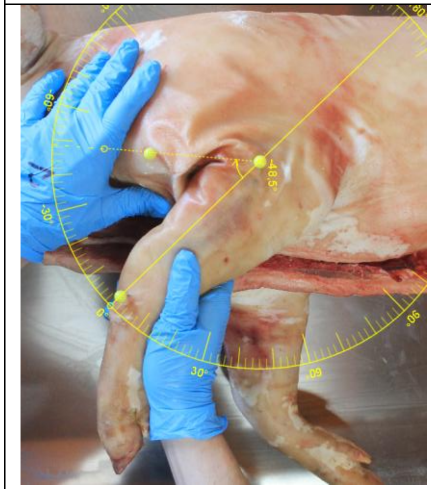	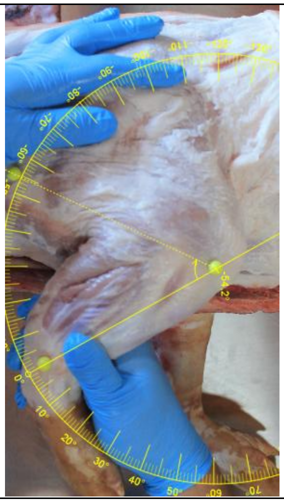	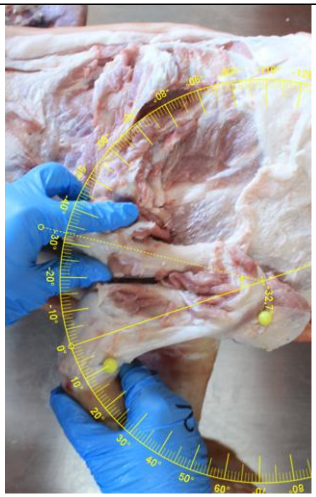	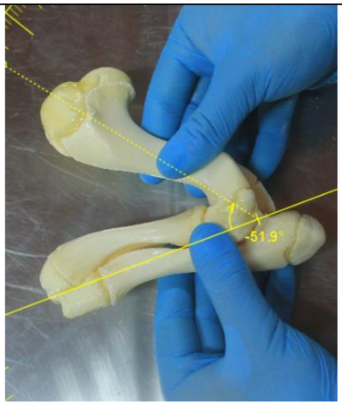
**Extension**			
**S + M+ CL + O**	**M+ CL + O**	**CL + O**	**O**
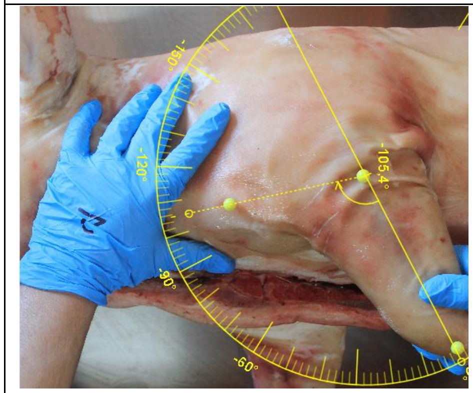	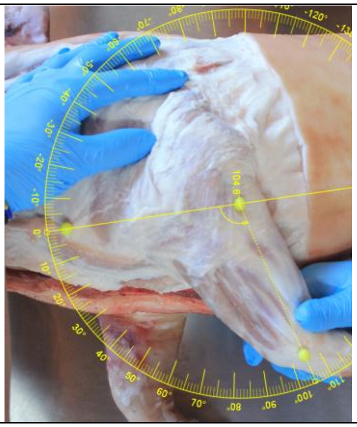	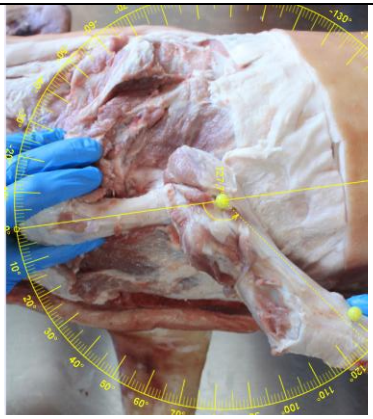	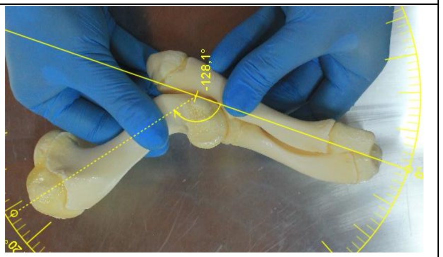

Wrist Flexion and Extension of Pig (*Sus scrofa*)

<table>
<tr><td colspan="4">Flexion</td></tr>
<tr><td>S + M+ CL + O</td><td>M+ CL + O</td><td>CL + O</td><td>O</td></tr>
<tr><td>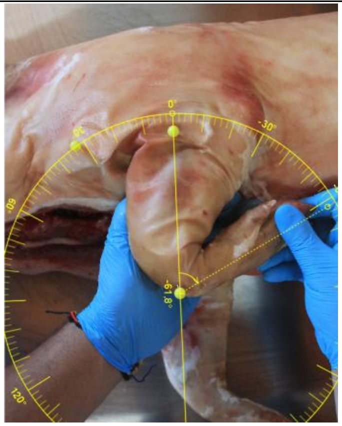</td><td>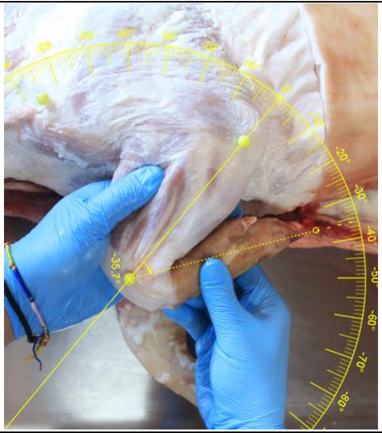</td><td>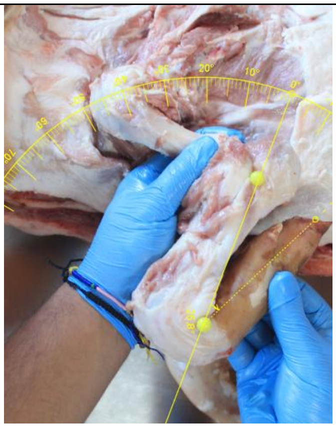</td><td>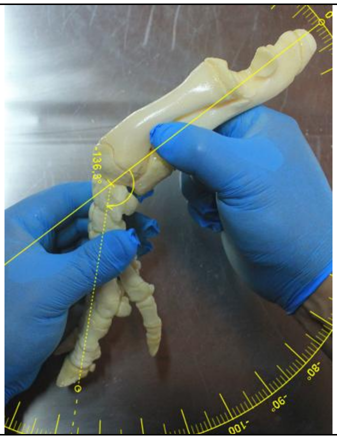</td></tr>
<tr><td colspan="4">Extension</td></tr>
<tr><td>S + M+ CL + O</td><td>M+ CL + O</td><td>CL + O</td><td>O</td></tr>
<tr><td>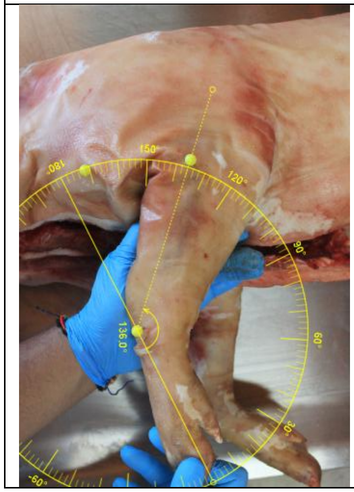</td><td>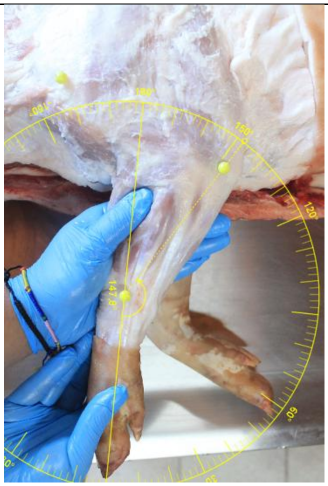</td><td>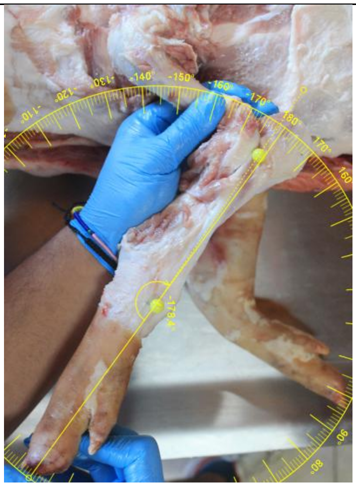</td><td>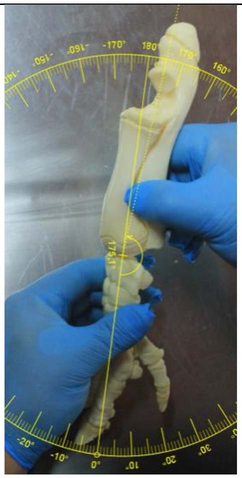</td></tr>
</table>

Hip Flexion and Extension of Pig (*Sus scrofa*)

**Flexion**			
**S + M+ CL + O**	**M+ CL + O**	**CL + O**	**O**
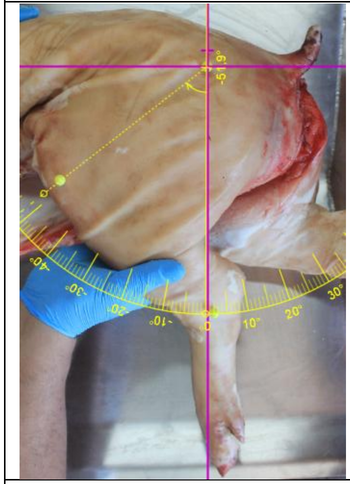	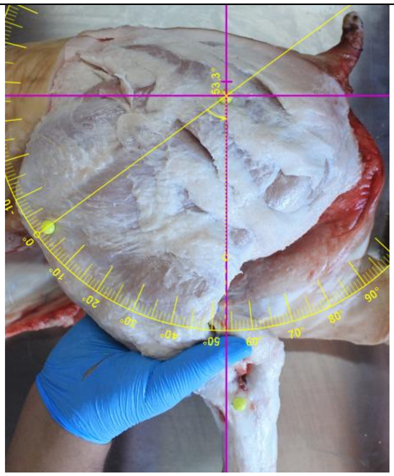	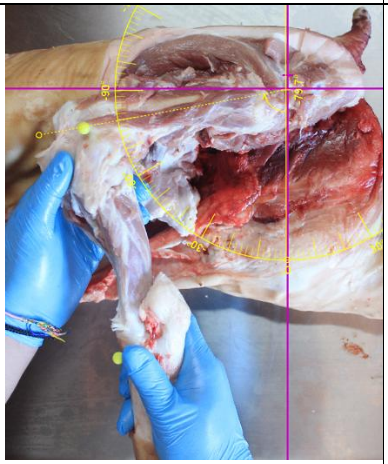	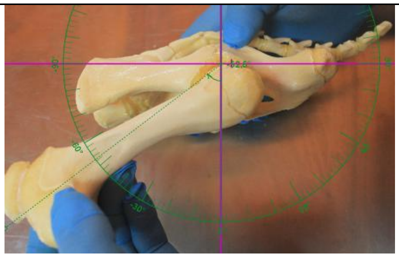
**Extension**			
**S + M+ CL + O**	**M+ CL + O**	**CL + O**	**O**
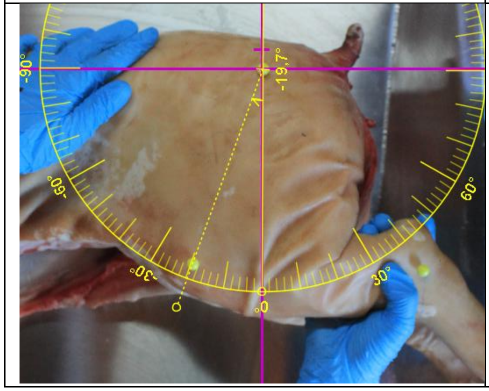	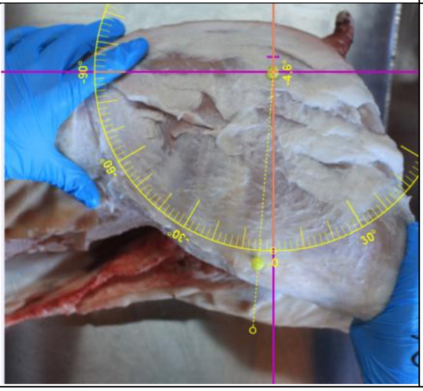	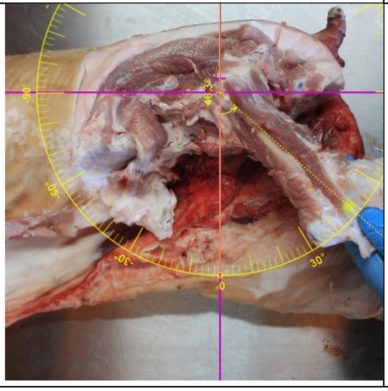	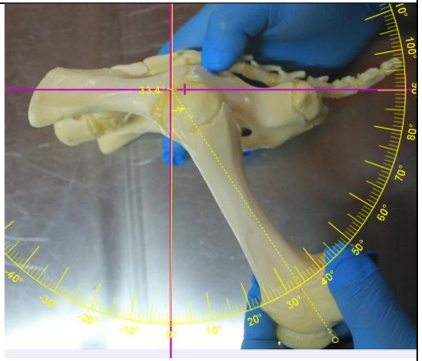

Knee Flexion and Extension of Pig (*Sus scrofa*)

**Flexion**			
**S + M+ CL + O**	**M+ CL + O**	**CL + O**	**O**
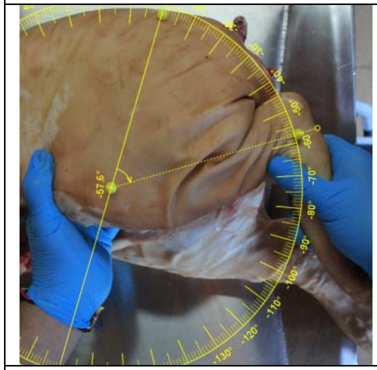	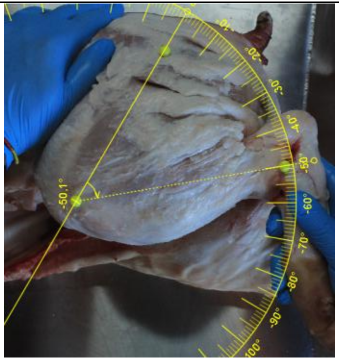	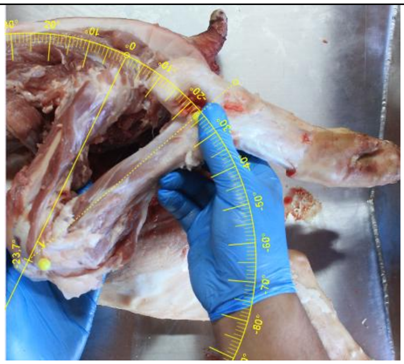	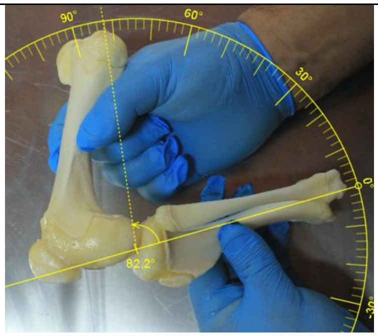
**Extension**			
**S + M+ CL + O**	**M+ CL + O**	**CL + O**	**O**
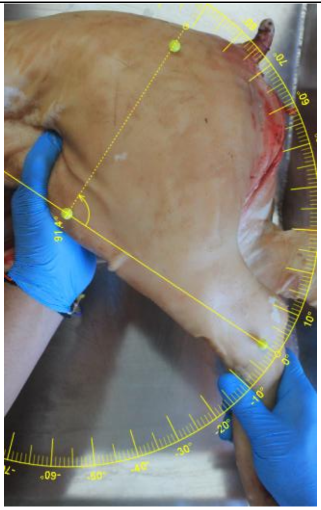	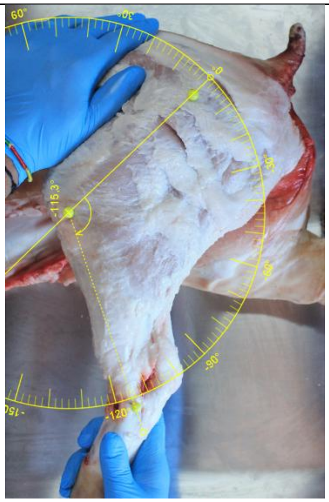	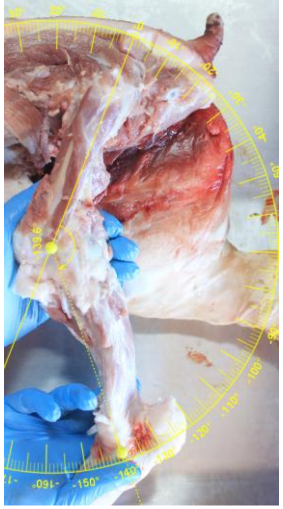	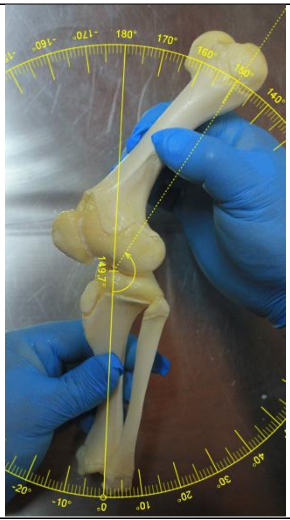

Ankle Flexion and Extension of Pig (*Sus scrofa*)

**Flexion**			
**S + M+ CL + O**	**M+ CL + O**	**CL + O**	**O**
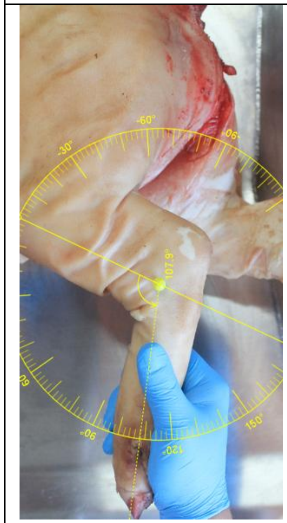	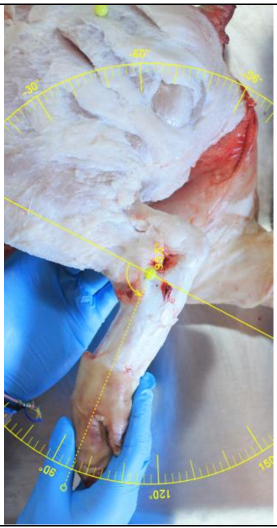	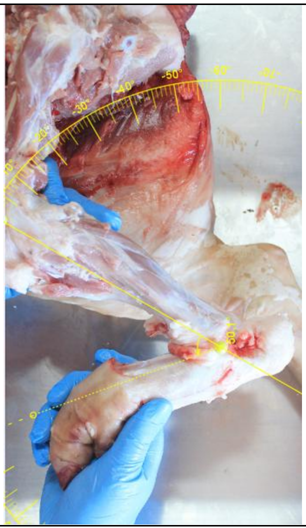	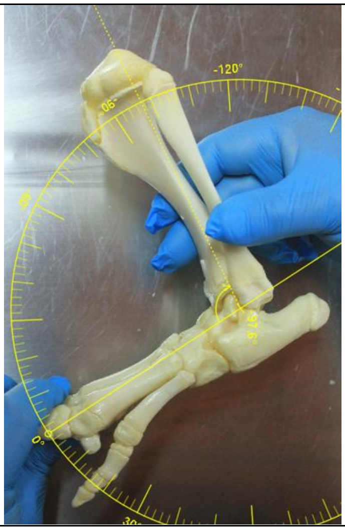
**Extension**			
**S + M+ CL + O**	**M+ CL + O**	**CL + O**	**O**
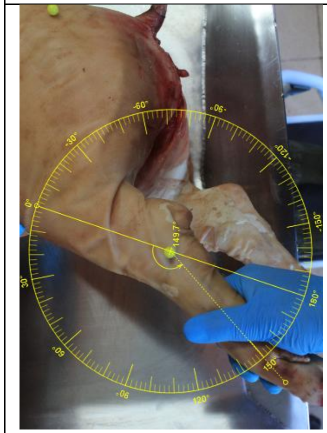	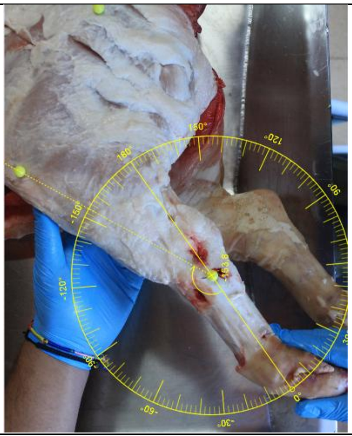	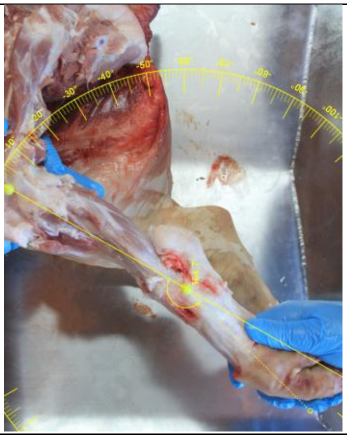	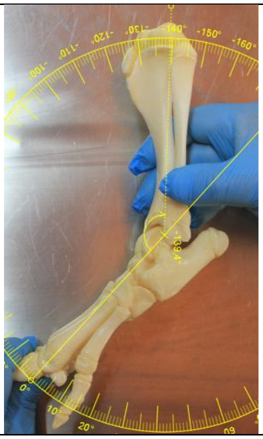
